# Humpback whale song recordings suggest common feeding ground occupation by multiple populations

**DOI:** 10.1038/s41598-021-98295-z

**Published:** 2021-09-22

**Authors:** Elena Schall, Karolin Thomisch, Olaf Boebel, Gabriele Gerlach, Sari Mangia Woods, Irene T. Roca, Ilse Van Opzeeland

**Affiliations:** 1 Alfred Wegener Institute for Polar and Marine Research, Bremerhaven, Germany; 2 Helmholtz Institute for Functional Marine Biodiversity, Carl Von Ossietzky University Oldenburg, Oldenburg, Germany; 3 Carl Von Ossietzky University Oldenburg, Oldenburg, Germany; 4 Marine Evolution and Conservation, Groningen Institute of Evolutionary Life Sciences, University of Groningen, Groningen, The Netherlands

**Keywords:** Conservation biology, Behavioural ecology, Marine biology

## Abstract

Humpback whale males are known to sing on their low-latitude breeding grounds, but it is well established that songs are also commonly produced ‘off-season’ on the feeding grounds or during migration. This opens exciting opportunities to investigate migratory aggregations, study humpback whale behavioral plasticity and potentially even assign individual singers to specific breeding grounds. In this study, we analyzed passive acoustic data from 13 recording positions and multiple years (2011–2018) within the Atlantic sector of the Southern Ocean (ASSO). Humpback whale song was detected at nine recording positions in five years. Most songs were recorded in May, austral fall, coinciding with the rapid increase in sea ice concentration at most recording positions. The spatio-temporal pattern in humpback whale singing activity on Southern Ocean feeding grounds is most likely shaped by local prey availability and humpback whale migratory strategies. Furthermore, the comparative analyses of song structures clearly show a differentiation of two song groups, of which one was solely recorded at the western edge of the ASSO and the other song group was recorded throughout the ASSO. This new finding suggests a common feeding ground occupation by multiple humpback whale populations in the ASSO, allowing for cultural and potentially even genetic exchange among populations.

## Introduction

Humpback whales annually undertake one of the longest mammalian migrations between their mid to high latitude feeding areas and low latitude breeding areas^1^. Various hypotheses on what drives baleen whale migration between such extremely spatially separated habitats have been put forward^2,3^, but to date, the reasons have not been understood entirely. On the breeding grounds, humpback whale sexual selection, copulation and parturition are presumed to take place^4–6^. Besides physical advertisement and intra/intersexual competition strategies (i.e., escorting of females and physical aggression among males)^4,6^, humpback whale males also perform acoustic displays in the form of songs^5,7^. Humpback whale song is speculated to fulfil a multi-purpose role within the species’ mating system, in many aspects comparable to bird song^5,8^. The majority of songs are therefore produced on the low-latitude breeding grounds, but ‘off-season’ song has also repeatedly been recorded along migration routes and on feeding grounds during different times of the year alongside recordings of social and feeding sounds^7,9–16^. Opportunistic singing outside the breeding grounds and/or season is interpreted as low-cost reproductive advertisement by males, although to date copulation has never been visually observed^14,17^.

Not much is known on which humpback whale stocks use which areas for feeding in the Southern Hemisphere^18,19^. Given that songs are breeding population-specific, the presence of song on the feeding grounds opens the possibility to assess breeding stock affiliation by comparative analyses of songs^5,7,15,20–22^. Male humpback whales on a specific breeding ground are known to converge closely on the same current rendition of song, termed song type^5,22–25^. Each song type is characterized by a distinct combination of themes, which in turn are built by the repetition of specific phrase types and each phrase type is composed of a unique combination of units^7,26^. Songs recorded on feeding grounds are composed of the same hierarchical structure as on the breeding grounds, although in some cases less complex song sequences or fragments of songs were registered^13,15,27–30^. The fact that humpback whales sing on the feeding grounds is thought to facilitate cultural transmission of new songs within the breeding population, but potentially also between different stocks^28^.

On Southern Hemisphere feeding grounds, the data on humpback whale song occurrence and dynamics are still limited both spatially and temporally. At the same time, information on stock distributions while on the feeding grounds is lacking, but crucial to management decisions on ecosystem and population conservation^18,19,31^. To date, two studies have presented song recordings from Antarctic waters comprising four days from two sites^13,20^. One further study collected acoustic data near a humpback whale ‘super-group’ off western South Africa and describes the song that was recorded there^32^. These studies showed that the identification and structural analysis of humpback whale song from austral feeding grounds can provide valuable information on humpback whale behavioural ecology and potentially even offer insight into the breeding stock origin of humpback whale males present in the feeding areas.

By analysing a passive acoustic data set spanning 13 recording positions deployed throughout the Atlantic sector of the Southern Ocean (ASSO) covering multiple years between 2011 and 2018, this study is the first to investigate the large-scale spatio-temporal patterns in humpback whale song presence and structure in the Southern Ocean. Humpback whale habitats within the ASSO are dominated by the seasonal fluctuations of sea ice concentration and extent which temporally allows access to large areas of open ocean (i.e., in summer) and restricts access in winter when the sea ice cover extents northward of 60°S^33^. In the scope of this study, we link humpback whale acoustic behaviour with sea ice dynamics in order to discuss potential drivers shaping the species’ acoustic behaviour on a Southern Ocean feeding ground. Furthermore, by assessing humpback whale song structure, we explore the comparability of feeding ground song with songs on the breeding grounds, the role of the ASSO as an alternative mating ground, and the potential mixing of multiple breeding populations in the ASSO feeding area.

## Results

In total, 186,074 h of recordings were processed, of which 4796 h were verified to contain humpback whale vocalizations (for details on data processing see the methods section at the end of this manuscript). From the latter 3239 h contained exclusively humpback whale social calls and the remaining hours contained songs. Songs were divided in two categories: the complex song (HWS1; songs organized in at least two different themes), which was found in 1127 h, and the preliminary song (HWS2; vocalization bouts which did not conform to the rule of the complex song category, but still formed at least three repeated phrases of the same phrase type) which was found in 430 h.

### Spatio-temporal pattern in song production

**Figure 1:**
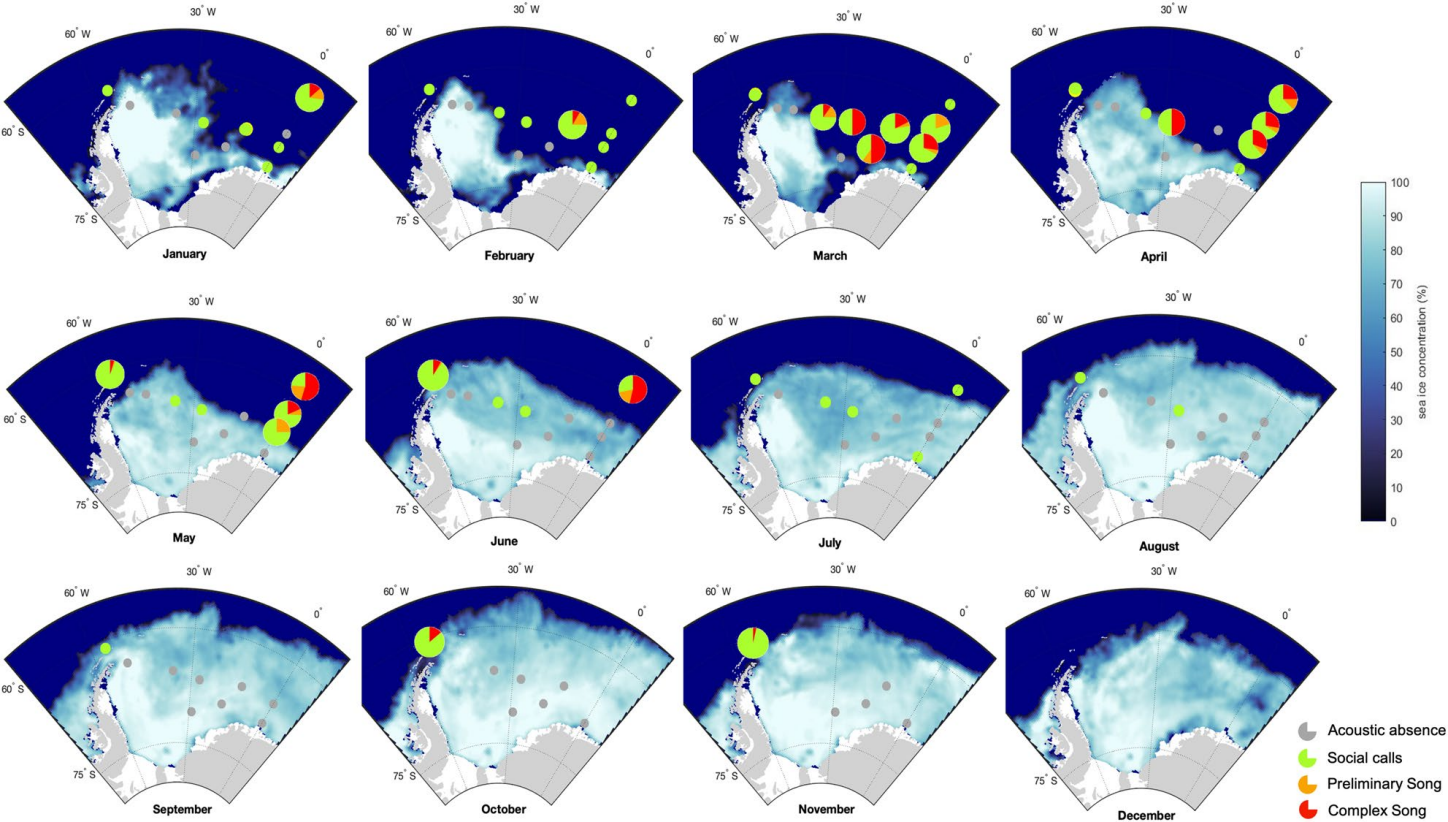
Proportion of social calls, preliminary song and complex song of humpback whales in the ASSO averaged per recording location and month for the year 2013. The monthly averaged sea ice concentrations are depicted at a 25 × 25 km resolution and maps were generated with M_MAP in MATLAB^95^. (See Schall et al.^40^ for details on acoustic presence).

**Figure 2:**
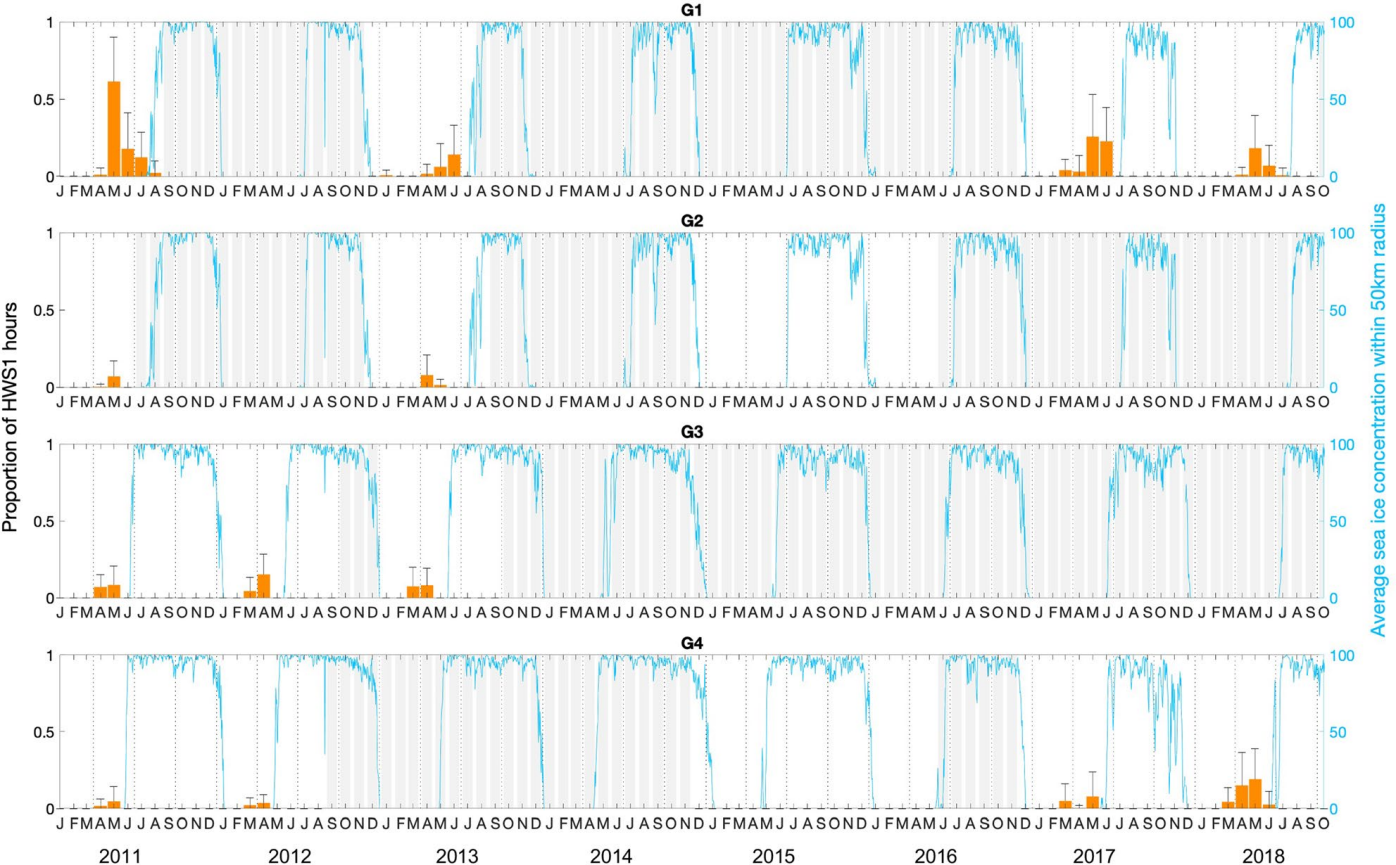
Proportion of HWS1 hours at the Greenwich Meridian averaged per month and recording location (G1–G4) from January 2011 until October 2018 (orange bars). Vertical error bars show the respective standard deviations and continuous grey bars represent months without recording data. The blue solid lines and the right y-axis depict the daily averaged sea ice concentration per location within a 50 km radius.

At ten out of the 13 recording locations, the acoustic presence of humpback whales (entailing the detection of any humpback whale vocalization, including social calls) included the presence of humpback whale song (Fig. 1). Songs were recorded in all years, except in 2015 and 2016 when recorders logged also almost no acoustic presence. The preliminary HWS2 was found in a similar spatio-temporal pattern as the complex HWS1, only in lower numbers. The earliest song of the season was detected at the recording position G1 on January 24, 2013 and the latest song of the season was detected at the same recording position on August 3, 2011 (Fig. 2). Song recordings were seasonally restricted to the summer and autumn months, except for the sporadic song recordings during spring 2013 off Elephant Island. Most songs were detected at the recording locations G1, G2, G3, and G4 on the Greenwich Meridian in the months April, May, and June (Figs. 1 and 2). During these months, songs were recorded continuously throughout the day or during random (even) hours of the day. March was the month when (complex) song was recorded at the most recording positions (i.e., at five positions). Summarizing all song recordings over years and positions, the number of hours containing humpback whale song is highest in May. This peak coincides with the rapid increase in sea ice concentration in late summer/autumn (Fig. 2). The first song recordings of the season were within 54 and 143 days after the sea ice concentration dropped below 15% (which defined the sea ice edge)^34^. The last song recordings of the season were maximally 12 days after the sea ice concentration exceeded 15%.

Of 77 individual singers (see methods section for details on the identification of individual singers), high quality sequences of complex song (i.e., signal-to-noise ratio ≥ 10 dB and at least two distinct themes discernible) were analysed in more detail to determine song structure (for details on song structure analysis see the methods section at the end of this manuscript). Measures of song session and song length (measured in number of units) did not show a clear trend in the course of the year or any trend along a latitude gradient (Supplementary Material 1: Table S1, Fig. S2). A slight increase of song session and song length could be observed from calendar day 120 to calendar day 182, with a maximum mean song session length of 1603 units and a maximum mean song length of 400.75 units on calendar day 182.

The level of agreement between the manual unit classification and the result of the supervised machine learning approach was high with a OOB misclassification rate of 16% indicating a robust differentiation of units, phrases, themes and songs (i.e., 62 phrase types; see Supplementary Material 2). Resulting measures of unit, phrase and song complexity (measured as number of unique unit and/or phrase types per song sequence) did not show a trend in the course of the year or along a latitude gradient (Supplementary Material 1: Table S1, Fig. S2). Different levels of complexity were almost equally distributed throughout time and across latitude.

### Song differentiation in the ASSO

**Figure 3:**
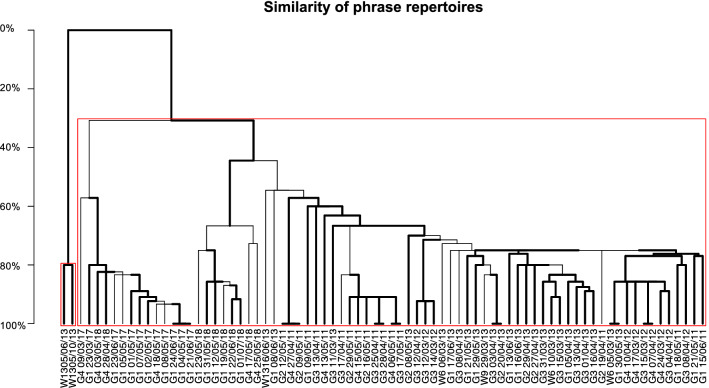
Bootstrapped dendrogram from hierachical clustering of set median song strings recorded at different locations and years, based on DCI analysis. Names on each branch belong to individual singers encoded with the name of the recording position (first 2–3 symbols, i.e., ‘W13’, ‘G4’,…) and the date of the recording (last 8 symbols, i.e., ‘05/06/13’, ‘05/10/13’,… representing dd/MM/YY). Bold lines indicate divisions that were likely to occur (i.e., AU > 95%) and red boxes indicate clusters which are strongly supported by the data.

The phrase repertoires of individual singers were strongly differentiated between the eastern and western edges of the ASSO as estimated by the bootstrapped hierarchical clustering of pairwise comparisons of phrase repertoires (compared with Dice Coincidence Index (DCI) calculated as the number of shared phrase types divided by the sum of the number of phrase types of each singer^35^). Two individuals recorded in autumn and spring 2013 off Elephant Island (i.e., singer IDs W1305/06/13 and W1305/10/13 representing location and date of recording; Table 1, Fig. 3) used a phrase repertoire which was completely different to all other phrase repertoires, whereas one individual recorded off Elephant Island did use a phrase repertoire which was similar to the phrase repertoires recorded on the eastern edge of the ASSO (i.e., W1316/06/13). All phrase repertoires from the eastern edge of the ASSO (i.e., Greenwich Meridian) and the central Weddell Sea (i.e., all Weddell Sea recorders except the recorder close to Elephant Island) were highly similar to each other depending on the year of recording. Phrase repertoires from the years 2011–2013 had the highest similarities to each other and phrase repertoires from 2017 and 2018 had variable similarities between 30 and 80%. The phrase repertoires from 2011–2013 and 2017/18 were at least 50% different. Some individual singers within the same recording year shared the phrase repertoire to a 100%.

**Table 1:** Set median theme sequences recorded at different locations and years in the Atlantic sector of the Southern Ocean.

Singer ID	Theme sequence	Singer ID	Theme sequence
G3 13/04/11	Cb Cc (17)	G3 13/04/13	Aa Ai (12)
G3 17/04/11	Ea Cb Ba Aa (5)	G3 16/04/13	Aa Ai An Ak Ac Ad (7)
G2 19/04/11	Ba Aa (4)	G2 20/04/13	Aa Ai Ac (7)
G3 25/04/11	Ea Cb Ca Cc Ba (3)	G2 27/04/13	Aa Ap Ai Aq Ac (3)
G4 27/04/11	Cb Cc (4)	G2 29/04/13	Aa Ac Ai Ak Ap (2)
G3 28/04/11	Cb Cc Ca Ba Ea (2)	G2 08/05/13	Ap Aa (10)
G4 06/05/11	Ea Cb Cc Ca (8)	G1 21/05/13	Aa Ai Ac (12)
G2 09/05/11	Cb Cc (3)	G1 29/05/13	Aa Ai Ac (10)
G1 09/05/11	Ba Ac Aa (4)	G1 30/05/13	Aa Ai Ac (2)
G2 12/05/11	Cb Cc (9)	W1305/06/13	Ga Ha Ec Ed Gb (7)
G4 13/05/11	Ba Ac (13)	G1 08/06/13	Cb Fa Ba Ca Aj Ak (2)
G4 15/05/11	Ea Cb Cc Aa (3)	G1 13/06/13	Aa Ac (16)
G2 16/05/11	Cb Cc Ba Aa (4)	G1 16/06/13	Aa Ai Ac Aq (22)
G3 17/05/11	Aa Ea Cb Cc Ca Ba (5)	W1316/06/13	Ai Ap (4)
G1 18/05/11	Ba Aa Ac Bb Ab (3)	G1 17/06/13	Ap Aa Ai (1)
G1 21/05/11	Ba Aa Ac (21)	W1305/10/13	Ed Gb Ga (4)
G2 29/05/11	Cb Cc (7)	G4 09/03/17	Df Ee (2)
G1 15/06/11	Cc Cb Ba Aa Ea (15)	G1 23/03/17	Bf Bd Bg Ee (1)
G3 12/03/12	Aa Ba (8)	G1 01/05/17	Bd Be Df Ee (21)
G3 14/03/12	Ac Aa Af Da De Ba (1)	G1 02/05/17	Bd Df Ee (5)
G3 15/03/12	Ac Aa Ba (2)	G1 04/05/17	Be Df Ee Bf (8)
G4 17/03/12	Aa Ai Ba (1)	G1 05/05/17	Bd Bg Df Ef Ee (1)
G4 24/03/12	Aa Ac Ba (4)	G1 07/05/17	Df Ee (10)
G3 04/04/12	Aa Ac Ba (8)	G1 08/05/17	Bg Df Ee (10)
G4 07/04/12	Aa Ac Ba (2)	G4 18/05/17	Df Bd Be Ee (11)
G3 08/04/12	Ba Aa (17)	G1 21/06/17	Bf Be Df (4)
G4 10/04/12	Aa Ai Aj Ba (3)	G1 23/06/17	Bd Be Df Ef (3)
G3 12/04/12	Ba Aa Af Da (7)	G1 24/06/17	Df Dg Bg (11)
W6 05/03/13	Aa Ai Ac (4)	G4 28/04/18	Bg Bd Be Df Ef Gd (4)
W6 06/03/13	Ad Aa Ac (3)	G4 03/05/18	Bd Df (12)
W6 10/03/13	Aa Ai Ak Ac (6)	G1 12/05/18	Gd Ge Gg Gh Ib (11)
G3 11/03/13	Ai Ac (4)	G4 17/05/18	Bh Bi (3)
G3 15/03/13	Ac An Ak (5)	G1 19/05/18	Gd Gf Gg Ge Bi Bb Bj (8)
W9 29/03/13	Ap Aa (12)	G1 23/05/18	Bi Bb Ib Bh Bc Gd Gh (2)
G3 31/03/13	Aa Ai Ak Ac (13)	G4 25/05/18	Gd Gf Gg Bh Bb Ba (2)
G3 01/04/13	Ac Aa Ai (10)	G1 31/05/18	Gd Ge (3)
G3 03/04/13	Aa Ai Aj Ac Ad (4)	G1 22/06/18	Gd Gg Bh Bi Bb (20)
G1 05/04/13	Ac Aq Aa Am Ai An Ak (24)	G1 01/07/18	Gd Gg Bh Bi Gh Bb Ge Gf Bc Bj Ib (4)
G3 08/04/13	Aq Aa Am Ai Ac (1)		

‘Singer IDs’ correspond to individual singers encoded with the name of the recording position (first 2–3 symbols, i.e., ‘W13’, ‘G4’,…) and the date of the recording (last 8 symbols, i.e., ‘05/06/13’, ‘05/10/13’,… representing dd/MM/YY). Theme sequence is encoded with phrase type names each composed of a uppercase and a lowercase letter (see Supplementary Material 2 for phrase type catalogue). Number in brackets is the number of theme sequences that was analyzed for each individual singer.

**Figure 4:**
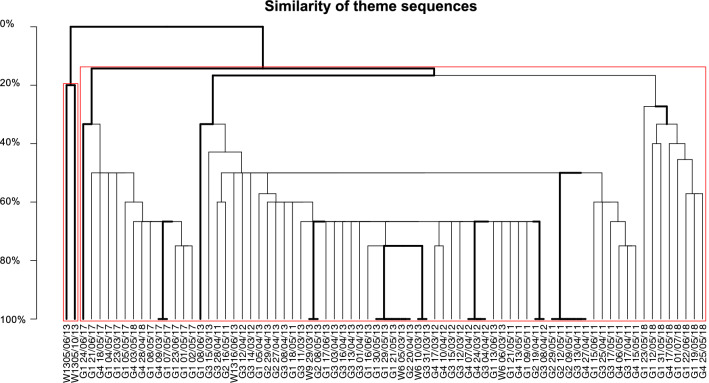
Bootstrapped dendrogram from hierachical clustering of set median song strings recorded at different locations and years, based on LSI analysis. Names on each branch belong to individual singers encoded with the name of the recording position (first 2–3 symbols, i.e., ‘W13’, ‘G4’,…) and the date of the recording (last 8 symbols, i.e., ‘05/06/13’, ‘05/10/13’,… representing dd/MM/YY). Bold lines indicate divisions that were likely to occur (i.e., AU > 95%) and red boxes indicate clusters which are strongly supported by the data.

The song structure in terms of theme order was again highly differentiated between the eastern and western edges of the ASSO as estimated by the bootstrapped hierarchical clustering of pairwise comparisons of theme sequences (compared with Levensthein Distance Similarity Index (LSI) calculated as the minimum number of insertions, deletions and substitutions required to change one string into the other divided by the length of the longer string^36^). Except for the two distinct individual singers from 2013 off Elephant Island (Table 1, Fig. 4), all other recorded song sequences from the years 2011, 2012, and 2013 were similar in structure, i.e., with similarities between 30 and 70%. Song sequences from 2017 and 2018 were only 20% similar to the song sequences from the other years and between the years 2017 and 2018 similarity was also low (i.e., 20%), except for two individual singers from 2018 which were recorded early in the season (i.e., G403/05/18 and G428/04/18).

## Discussion

### Spatio-temporal pattern in song production

The present study is the first record of the large-scale occurrence of humpback whale song in the ASSO. Humpback whale song was recorded at nine of the 13 recording positions and multiple years of song recordings were registered in the course of this study. Our data was able to show for the first time that singing activities occur over a large spatio-temporal scale on the feeding grounds in the Southern Ocean. 2015 and 2016 were the only years with no humpback whale song recordings, which is probably related to the physical absence of humpback whales from the area in these years due to unfavourable environmental conditions^37^.

The presence and absence of humpback whale song on the feeding ground might be directly determined by local prey availability, as whales might be spending more time searching for food when local prey abundance is low, negatively affecting the likelihood of displaying singing behaviour. In zebra finches (*Taeniopygia guttata*), experiments showed that singing rates decreased when the prey availability was reduced^38^. Both changes in body condition and time budget available for acoustic displays were suggested as two possible connections between the availability of food and singing behaviour. It can therefore not be ruled out that humpback whales were present in the area around the Greenwich Meridian in 2015 and 2016, but that individuals produced no or very little calls. Schall, et al.^37^ documented limited acoustic presence of humpback whales (only few social calls during single days) in 2015 and 2016 at the Greenwich Meridian and suggested that climate oscillations possibly negatively affect krill productivity. Therefore, whales might need to spend more time foraging in the ASSO or forage elsewhere to fulfil their energetic needs and skip singing before migration in the ASSO during these years. This reduction of singing behaviour in humpback whales due to environmental factors (e.g., temperature, wind, sea ice condition, location of oceanographic fronts) could also explain the small inter-annual differences in the amount of song recorded among the years 2011, 2012, 2013, 2017, and 2018. Spatio-temporal patterns of song production are probably linked to large-scale ecological (e.g., prey) and environmental (e.g., temperature) variabilities, which has also been suggested for Northern Hemisphere humpback whales^39^.

Spatially, humpback whale song was found at all recording positions where acoustic presence was registered except the southernmost recording position at the Greenwich Meridian (G5^40^). This recording position is the closest to the Antarctic continent among all analysed recording positions and most of the time of the year it is covered by sea ice. The environmental conditions at this recording position are very similar to the conditions at the coastal recording station PALAOA, where similarly only humpback whale social calls were recorded during many months of the years 2008 and 2009, but no humpback whale songs were registered^41^. These combined results potentially support previous suggestions that the habitat close to the continent with an often dense ice cover might only be used by females and/or immature whales residing here throughout winter to presumably improve body condition^41,42^. This migratory-segregation depending on sex, age, and reproductive status in humpback whales^43^ possibly also explains the detection of social calls at other recording positions during the winter months when at the same time no humpback whale songs were recorded.

The detections of humpback whale songs were in general strongly seasonal. Male song production increased with the end of the summer/beginning of autumn (i.e., pre-migration singing, similar as observed in the Northern Hemisphere^16,21,44^) alongside with rapidly increasing sea ice concentrations. Humpback whale males seem to travel as far south as the sea ice retreats in summer and also adapt their northward migration to the expansion of the sea ice in autumn^41,45,46^. To optimize access to females, sexually mature males may not travel as far into the ice compared to females or immature males, to ensure their in-time arrival at the breeding grounds which may have reproductive advantages^42,47^. While the males still roam on the feeding grounds, they already commence the so-called (pre-breeding) shoulder season with the start of song production^13,14,16,20^. In other baleen whale species, song production has also been documented to occur outside the breeding area and season^48–53^, but the functionality of “off-season” song remains unknown. Similarly, some humpback whale males still sing when they arrive at the feeding ground in spring (during the post-breeding shoulder season)^14,27,44^, which in the case of the ASSO was only observed at Elephant Island (W13). In tropical birds, the year-round production of song is related to territorial defense and is thought to play a role in interspecific communication^54,55^. Singing activities in humpback whale males are thought to be triggered by elevated testosterone levels which slowly increase during the end of summer and decreases in spring^5,56^. Additionally, sexually mature males might also start singing when nutritional status allows singing activities during breaks from feeding. In song birds, the nutritional status has been shown to be a crucial factor affecting the amount of singing^57,58^. For example, male Bengalese finches showed higher song output including higher rates of singing and longer songs when receiving a high-nutrition diet compared with males receiving a moderate-nutrition diet^57^. The length of the pre-breeding shoulder season in our data (up to 5 months) indicates that humpback whale males during this time mix feeding and singing behaviour on a regular basis^13,59^. Early whaling studies showed that the timing of conception in Southern Hemisphere humpback whales ranged between June and October^60,61^. If the assumption that singing in humpback whales is primarily related to breeding activities is correct^5^, the ASSO might serve as an alternative breeding ground for the part of the population which skips migration.

Feeding grounds and pre-breeding shoulder seasons have been suggested to be the place and the time for the annual events of humpback whale song innovation^15,62,63^. Our data do not suggest a clear sign of song development on the feeding ground. The less complex preliminary song category (HWS2) was detected in lower numbers than the complex song category (HWS1) during almost all months when humpback whale songs were recorded. Additionally, the analysis on song complexity and length suggests that songs recorded on the ASSO feeding ground do not get more elaborate in the course of the season, only a slight increase in song and session length was detected. McSweeney, et al.^15^ discovered that songs on the feeding ground were shorter than the comparable songs on the breeding ground. However, the sample size in this study was very small and thus the increase in session/song length in the course of the season on the feeding ground potentially remained undetected. Vu, et al.^14^ also detected an increase in session length in autumn and suggested a connection between the amount of singing activity and the testosterone level. Our results indicate that this connection could also be true for singing activity on Southern Ocean feeding grounds. Song complexity and the process of developing the complex breeding ground song on the feeding ground, in contrast, seems not to be connected with the elevation of testosterone levels. Instead, humpback whale males might start singing the song from the previous breeding season and change or adapt random themes in the course of the season until the new song is formed^15,20^. However, it cannot be ruled out that other measures for song complexity as a condensed ‘complexity score’ or phrase transition patterns may have shown trends over the course of a season^28,64^. The change or adaptation of themes is probably a product of cultural transmission of songs among and within different breeding populations while whales visit common feeding areas^9,20,62^. The production of song on the ASSO feeding grounds could therefore serve the facilitation of this cultural transmission to increase the chances of reproduction on the breeding grounds by singing a newly innovated version of song and/or could have direct benefits to the reproductive success of males in place.

### Song differentiation in the ASSO

Although humpback whale males might not sing the fully developed breeding ground song on the feeding ground, our data suggest a clear differentiation of two distinct song groups, which most likely belong to (at least) two distinct humpback whale breeding stocks. The parallel presence of two distinct song groups in the ASSO demonstrates its ecological significance for cultural and maybe even genetic exchange among humpback whale breeding stocks in this area. One song group was recorded in 2013 exclusively at the western edge of the ASSO, north of the Antarctic Peninsula, and close to the coast of Elephant Island. The other song group was recorded throughout the ASSO from 2011 to 2018. These two song groups were completely different both in phrase repertoire and theme sequence. The clear result of higher differentiation between these two groups than among years indicates that at least two different breeding populations visit the ASSO as a feeding area. The fact that song sequences of both song groups were recorded off Elephant Island additionally indicates that the distinct breeding populations spatially overlap in their distribution on the feeding ground. At least four distinct breeding stocks are in spatial vicinity to the ASSO on the longitude scale: Breeding stock G in the eastern South Pacific, breeding stock A in the western South Atlantic, breeding stock B in the eastern South Atlantic, and breeding stock C in the western Indian Ocean^18^. Humpback whales from the breeding stock G are thought to occupy the Antarctic management area I (120–60°W) as a feeding ground, which has been proven by genetic and Photo-ID studies^65,66^. A circumpolar study on humpback whale genetics has shown that humpback whales from the Antarctic management area I are highly differentiated from all other management areas (except for samples collected close to management area I in management area II; 60°W–0)^67^. The two song sequences that were strongly different from the rest of the song sequences recorded during this study were recorded on the border between management area I and II, which makes it likely that this song group stems from a South Pacific breeding stock. The second song group including the majority of the song sequences recorded during this study probably stems from a South Atlantic breeding stock or could also be related to an Indian Ocean breeding stock. Previous studies have shown that songs from breeding stocks A, B, and C often show similarities both in repertoire as well as structure^68–70^. Satellite tagging studies have shown that humpback whales from breeding stock A and B both migrate to the eastern part of the South Atlantic^71,72^ and might therefore both contribute to the songs recorded in this study. Single song phrases detected in this study were also documented for song sequences recorded off the Western Cape of South Africa^12,32^. In order to fully understand the eventual sharing of common feeding areas among humpback whales from different breeding stocks and the cultural transmission of song among them, further comparative analyses of songs from the breeding grounds and the ASSO are necessary.

### Conclusions and outlook

The ASSO forms an important summer feeding habitat for various baleen whale species and different studies have also shown its importance as an overwintering ground^40,41,49,73^. The first evidence of humpback whale song over a large spatio-temporal scale furthermore proves the additional importance of the ASSO for reproductive activities. The distinct timing of song occurrence at the eastern and western edges of the ASSO together with the identification of two different song groups in these two regions indicates that at least two different breeding stocks of humpback whales use the ASSO for feeding and reproduction. Comparative song analyses including songs from the ASSO as well as songs from the different breeding stocks are planned to gather more detailed information on how the occupation of this large feeding area in the Southern Ocean connects to the acoustic recordings of humpback whale songs from lower latitudes. The identification of crucial habitats for migratory baleen whales, as well as, the linkages between breeding and feeding grounds is of key importance for stock management and the planning of large-scale marine protected areas^19,31^.

## Methods

### Data and processing

**Figure 5:**
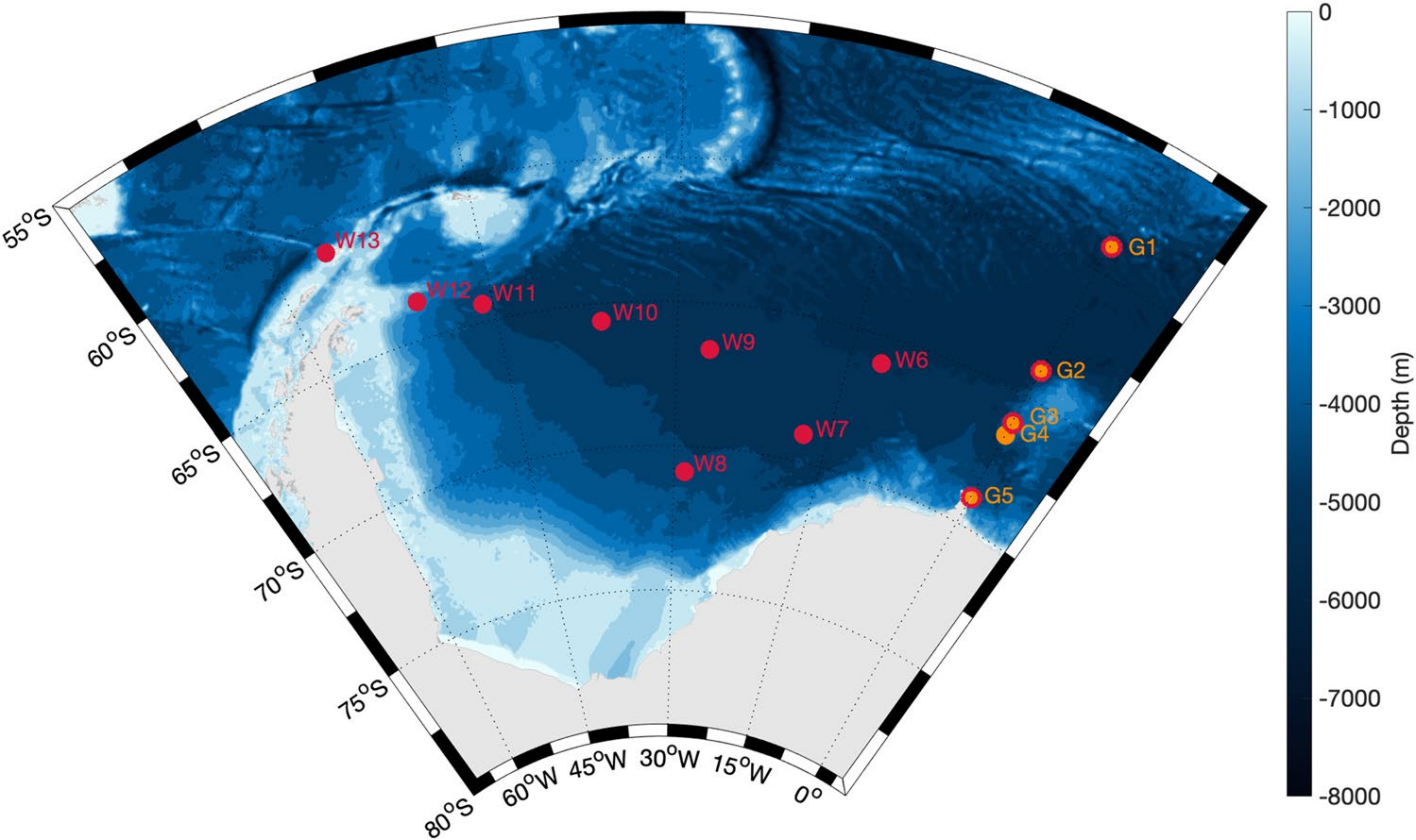
Mooring positions included in this study. Mooring positions marked in orange and labelled with the prefix ‘G’ in the name are part of the multi-year Greenwich dataset (2010–2018). Mooring positions in red and labelled with the prefix ‘W’ in the name are part of the single-year Weddell dataset (2013). Mooring positions which are marked in orange and red are part of both datasets. Map was generated with M_MAP in MATLAB^95^.

We investigated humpback whale acoustic behaviour using data from 13 recording positions throughout the ASSO (Fig. 5) which recorded in different periods between 2011 and 2018 (five recording positions form the multi-year Greenwich dataset and eight recording positions form the single-year Weddell dataset; Supplementary Material 1: Fig. S1). Passive acoustic recordings were obtained using SonoVaults (Develogic GmbH, Hamburg) operated on a continuous recording scheme and with a sampling rate of 5333 to 9600 Hz^74^.

All available passive acoustic data were processed by the ‘Low Frequency Detection and Classification System’ (LFDCS) developed by^75^ and a custom-made acoustic-context filter to detect humpback whale acoustic presence at an hourly basis. LFDCS was set up with a customized call library based on the most common vocalization types of humpback whales and other acoustically abundant Antarctic marine mammal species (i.e., Antarctic minke whale (*Balaenoptera bonaerensis*), killer whale (*Orcinus orca*), Weddell seal (*Leptonychotes weddellii*), crabeater seal (*Lobodon carcinophaga*), leopard seal (*Hydrurga leptonyx*), and Ross seal (*Ommatophoca rossii*))^76–81^. Parameter settings and thresholds of LFDCS and the acoustic context filter were tuned employing multiple test datasets to optimize the automatic detection of humpback whale vocalizations to the requirements of this study. Detailed information on set up and test runs of the automatic detection process are provided in Schall, et al.^40^.

The sea ice concentration data used for this study were extracted from: a combination of satellite sensor data from the Nimbus-7 Scanning Multichannel Microwave Radiometer (SMMR), the Defense Meteorological Satellite Program (DMSP) -F8, -F11 and -F13 Special Sensor Microwave/Im rs (SSM/Is), and the DMSP-F17 Special Sensor Microwave Imager/Sounder (SSMIS), with a grid size of 25 km^82^ and the satellite images from the Advanced Microwave Scanning Radiometer for EOS (AMSR-E) satellite sensor with a grid size of 6.25 km^33^. The data were used to calculate the daily sea-ice concentration of the area within 50 km radius around each recording location of the *Greenwich* dataset in MATLAB. Additionally, the data were used to calculate monthly averages of sea-ice concentrations for the *ASSO* and plotted as maps with the Antarctic Mapping Tools and Daily Antarctic Sea Ice Concentration packages in MATLAB^83,84^.

### Song presence

**Figure 6:**
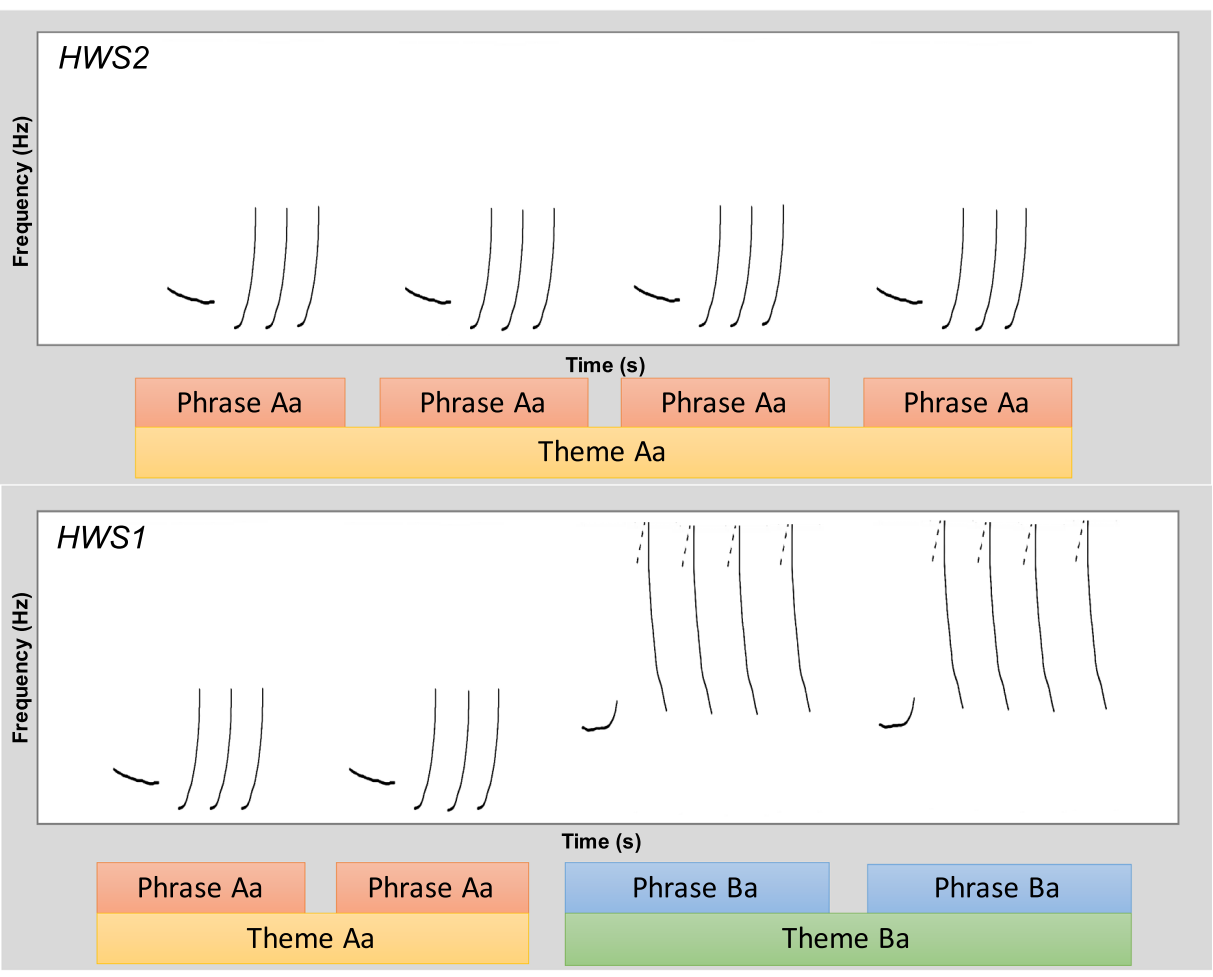
Schematic illustration of spectrogram visualizations of the preliminary humpback whale song 2 (HWS2) and complex humpback whale song 1 (HWS1) categories. HWS2 is defined as a vocalization sequence organized in at least three repeated, similar phrases and HWS1 is defined as a vocalization sequence organized in at least two different themes (see^26^ for details on phrase and theme delineation).

Even hours with presumed humpback whale acoustic presence (i.e., hours 0, 2, 4, 6, 8, 10, 12, 14, 16, 18, 20, 22 indicated by the automatic detector) were revised visually and aurally for the presence of humpback whale vocalizations by creating spectrograms in Raven Pro 1.5 (Hann Window, 1025–1790 window size, 80% overlap, 2048 DFT size; Bioacoustics Research Program 2014). Spectrograms were scanned for humpback whale vocalizations by viewing 60 s windows from 0 to 1.80 kHz. Hours with confirmed humpback whale acoustic presence were separated in hours with humpback whale social calls and hours with humpback whale song, applying guidelines from Cholewiak, et al.^26^. Hours with humpback whale song were further divided into two song categories: the preliminary song category and the complex song category. Humpback whale vocalizations that were organized in at least two different themes were classified as the complex song category 1 (*humpback whale song 1*; HWS1; Fig. 6). If humpback whale vocalization bouts did not conform to the rule of the complex song category, but still formed at least three repeated phrases of the same phrase type, the respective hour was classified as the preliminary song category 2 (*humpback whale song 2*; HWS2; Fig. 6).

### Song sequence analysis

Song sequences of humpback whales in the ASSO were investigated and catalogued by analysing all even hours with high quality complex songs (i.e., signal-to-noise ratio ≥ 10 dB and at least two distinct themes discernible). Both the preceding and succeeding odd hours to the respective analysed hour were also included in the analysis if those also contained high quality song sequences. Humpback whale vocalizations were manually logged within the spectrograms in Raven Pro (with identical spectrogram settings). Logged calls were manually classified into distinct unit types (call types: CT followed by a number) according to the following criteria: (1) differentiation of tonal or broadband characteristics, (2) duration, (3) frequency range and (4) time–frequency slope. Within a humpback whale song sequence, phrases were logged and classified according to unit repetition following Cholewiak, et al.^26^ recommendations. Phrase types were identified with an uppercase letter (indicating the 1^st^ unit type), a lowercase letter (indicating the combination of following unit types) and a sequence of numbers (indicating the number of repetitions of each unit) in order to be able to breakdown to the original unit sequence in the downstream analysis process.

The manual subjective analysis of unit and phrase repertoire was tested in terms of robustness by applying an automated classification approach to a subset of units (i.e., 436 exemplar units with at least 20 exemplars per unit type). We computed 44 different acoustic metrics for every extracted unit (i.e., 3 s sound file decimated to 5000 Hz to ensure comparability). The 44 metrics can be described as belonging to either of these three categories: (1) indices based on different algorithms to compute acoustic complexity, entropy or diversity (acoustic indices); (2) metrics measuring amplitude or background patterns (energy metrics); and (3) metrics computing ratios between acoustic activity over time and frequency bands (ratio metrics). Details on the acoustic metrices used and the process of computation for the 436 sound examples can be found in Schall, et al.^85^. The 44 acoustic metrices for each extracted unit were used in a supervised machine learning approach (i.e., random forest, see Schall, et al.^85^ for details) to discriminate between manually classified unit types and the automatic classification accuracy was assessed with the general ‘Out-of-bag’ (OOB) misclassification rate.

### Song structure, length and complexity

Registered song sequences were allocated to presumed individual singers in order to assess inter-individual variation in song sequences. Due to the nature of our single sensor autonomous recordings, song sequences cannot be attributed to individual calling males. Therefore, the following assumptions were made to differentiate among individual singers. Firstly, recordings of humpback whales at the distinct recording positions and at a specific point in time, were assumed to be distinct humpback whale individuals. Recording positions were situated at geographic distances of more than 200 km (except for the recording positions G3 and G4) which a humpback whale with an average swimming speed of 4 km/h^10^ is unlikely to travel within 24 h. Second, recordings of humpback whale song, between which more than 24 h had passed were assumed to belong to different individual singers due to the estimated travel rates of 17 to 75 km/day in humpback whales on an Antarctic feeding ground^86^.

Furthermore, for the following quantitative comparisons of song length, complexity, repertoire and structure, song sequences of individual singers were separated into song sessions and songs. Song sessions are commonly defined as all song elements sung until a gap of silence of more than one minute occurs^7,26^. The definition of the start and end of an explicit song can however be problematic due to the numerous distinct attempts defining a song in different studies^26^. Inspecting our song sequence data for common patterns, the most sensible definition for song in the ASSO seemed to be the complete rendition of all unique theme types per song sequence to form an explicit humpback whale song^26^.

To quantitatively compare the elaborateness (including complexity and length) of song per time of the year and latitude, two measures of length and three measures of complexity were included in the analyses. The length of song sessions and songs was measured as the number of vocalization units per sequence. Session and song length were averaged per individual singer and standard deviations were calculated. Furthermore, three measures of unit and phrase complexity were adapted from studies on bird song^87–90^. Unit complexity was defined as the number of unique unit types divided by the total number of units per song. Phrase complexity was defined as the number of unique phrase types divided by the total number of phrases per song. To adapt an overall measure of song complexity^64,89,90^, the unit complexity was multiplied by phrase complexity. The correlation between measures of song elaborateness and the time of year and latitude was assessed with the calculation of Pearson correlation coefficients.

### Song repertoire and structure comparison

The phrase repertoire of all individual singers was compared by applying the Dice Coincidence Index (DCI) with a custom-written script in R^35,91^:

$$DCI=2A/(B+C)$$

with *A* being the number of shared phrase types between a pair of singers, *B* and *C* being the number of phrase types of each singer, respectively. The resulting similarity matrix was supplied to a hierarchical cluster analysis in R^91^ using the “nearest neighbour” method and the output was visualized in a dendrogram. Hierarchical clustering was bootstrapped (1000 times) with the R function ‘pvclust’^92^ to generate approximate unbiased (AU) values with AU values exceeding 95% indicating dendrogram divisions that are likely to occur.

To compare the song structure among individual singers the sequences of phrases were transcribed to sequences of themes (i.e., ignoring the repetition of phrases) and a set median string was chosen for each individual singer. The set median string was defined as the sequence of themes which had the highest similarity to all sequences of themes of a given set, in this case, all songs recorded within a single 24-h window at one recording position. The similarity between sequences was calculated by applying the Levenshtein Distance Similarity Index (LSI) in MATLAB^36,93^:

$$LSI\left(a,b\right)=1-{\text{min}}(I+D+S)/{\text{max}}[L\left(a\right),L\left(b\right)],$$

with *a* and *b* being the two theme sequences, *I* being insertions, *D* being deletions, *S* being substitutions and *L* being the length of the respective sequence. In the following, the set median strings of all individual singers were compared by applying the LSI to pairs of individuals with the R function ‘stringdist’^94^. The resulting similarity matrix was supplied to a hierarchical cluster analysis using the “nearest neighbour” method, the output was visualized in a dendrogram, and hierarchical clustering was bootstrapped (1000 times)^91,92^.

## Supplementary Information

Supplementary Information 1.

Supplementary Information 2.

## Data Availability

Analyses reported in this article can be reproduced using the data provided by Schall (2021) at Data Dryad: https://datadryad.org/stash/share/tCp5x14Xl3xGdFHS36eonq7ENNXyVT832_jv5-n_xxA.
