# Supplementary material for: Humpback whale song recordings suggest common feeding ground occupation by multiple populations
Source: Sci Rep. 2021 Sep 22;11:18806. doi: 10.1038/s41598-021-98295-z (PMC8458523; doi:10.1038/s41598-021-98295-z)
Supplement: Supplementary file 1 — Supplementary Information 1. [file 41598_2021_98295_MOESM1_ESM.pdf]

Humpback whale song recordings suggest common feeding ground occupation by multiple populations

Authors

Elena Schall, Karolin Thomisch, Olaf Boebel, Gabriele Gerlach, Sari Mangia Woods, Irene Roca, Ilse Van Opzeeland

-Supplementary Material 1-

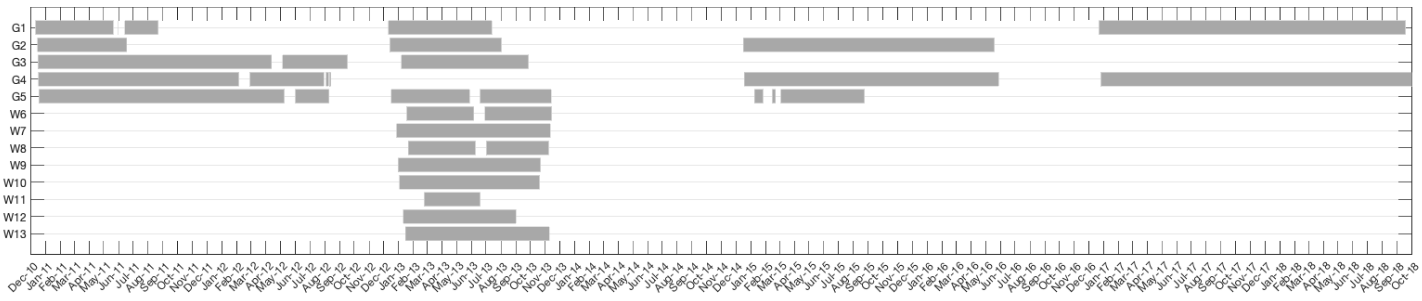

Figure S1. Timeline of acoustic recordings taken at the 13 recording locations of the Greenwich and Weddell datasets between 2010 and 2018. Mooring positions with the prefix 'G' in the name are assigned to the Greenwich dataset. Mooring positions with the prefix 'W' in the name are assigned to the Weddell dataset.

Table S1. Average (+/- standard deviation) session length, song length, unit complexity, phrase complexity, and song complexity per latitude of the recording location. Pearson correlation coefficient (r) for each measure against latitude is provided in the last row.

**Latitude**	**Session length**	**Song length**	**Unit complexity**	**Phrase complexity**	**Song complexity**
-59.05 (G1)	107.36 +/- 100.90	71.65 +/- 74.83	0.12 +/- 0.08	0.32 +/- 0.17	0.05 +/- 0.05
-61.02 (W13)	50.22 +/- 22.81	40.50 +/- 22.67	0.15 +/- 0.06	0.43 +/- 0.21	0.07 +/- 0.06
-64.00 (G2)	85.04 +/- 101.56	49.63 +/- 81.44	0.23 +/- 0.18	0.49 +/- 0.16	0.13 +/- 0.12
-65.97 (W9)	66.67 +/- 28.78	46.15 +/- 26.87	0.12 +/- 0.09	0.43 +/- 0.19	0.06 +/- 0.06
-66.03 (W6)	69.64 +/- 40.45	40.97 +/- 31.04	0.17 +/- 0.11	0.43 +/- 0.16	0.08 +/- 0.07
-66.51 (G3)	84.71 +/- 74.18	56.07 +/- 49.05	0.13 +/- 0.08	0.33 +/- 0.13	0.05 +/- 0.05
-66.61 (G4)	42.88 +/- 14.12	36.44 +/- 19.19	0.14 +/- 0.06	0.50 +/- 0.16	0.08 +/- 0.05
r	0.42	0.48	0.18	-0.54	0.11

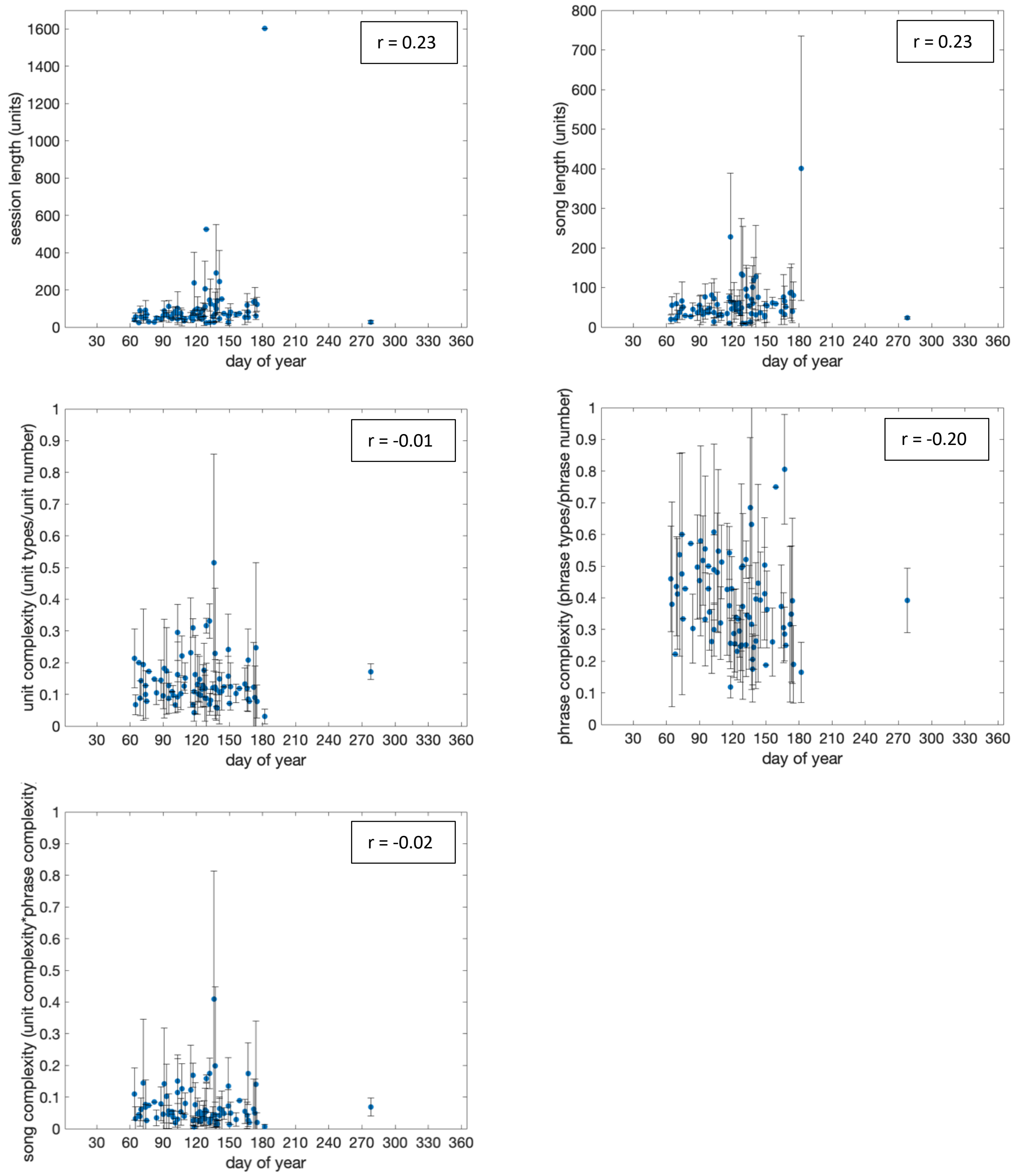

Figure S2. The five measures of song elaborateness (two for length and three for complexity) were plotted against day of the year pooled for all recording locations and years. Dots represent averages and error bars represent standard deviations. Pearson correlation coefficient (r) is provided in upper right corner of each plot.
