# Supplementary material for: Humpback whale song recordings suggest common feeding ground occupation by multiple populations
Source: Sci Rep. 2021 Sep 22;11:18806. doi: 10.1038/s41598-021-98295-z (PMC8458523; doi:10.1038/s41598-021-98295-z)
Supplement: Supplementary file 2 — Supplementary Information 2. [file 41598_2021_98295_MOESM2_ESM.pdf]

Humpback whale song recordings suggest common feeding ground occupation by multiple populations

Authors

Elena Schall, Karolin Thomisch, Olaf Boebel, Gabriele Gerlach, Sari Mangia Woods, Irene Roca, Ilse Van Opzeeland

-Supplementary Material 2-
Humpback whale call and phrase type catalogue - ASSO

- Call Types -

Representation of the call types used as song units.

Name	Example	Description
CT1 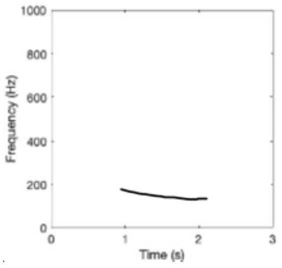	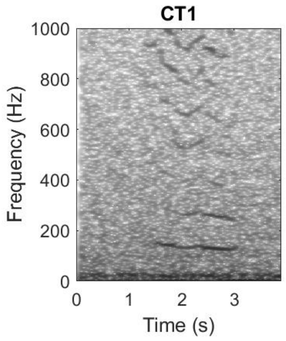	- tonal<br>- Fundamental 100-300Hz<br>- 1-2s<br>- Constant frequency contour<br>- Slightly downswept
CT3 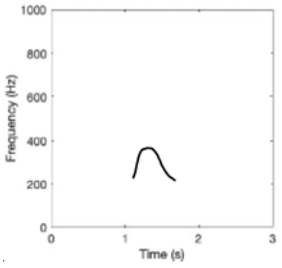	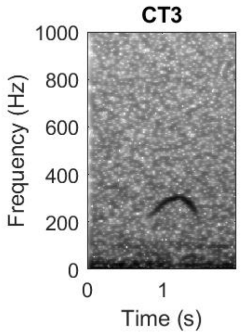	- tonal<br>- Fundamental 100-300Hz<br>- 0.5-1s<br>- Up-downsweep<br>- Sometimes up- or downsweep dominates

CT4 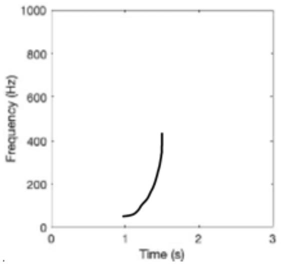	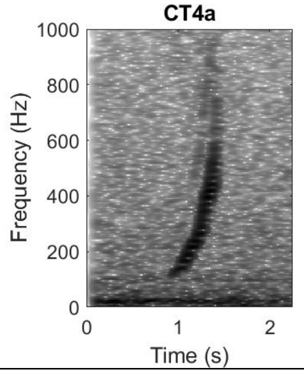	- tonal<br>- Fundamental 80-800Hz<br>- 0.3-0.7s<br>- Upsweep<br>- Sometimes almost pulsed
CT5 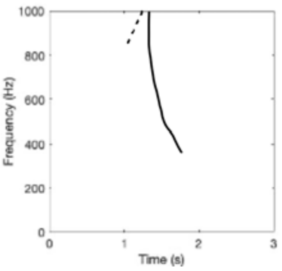	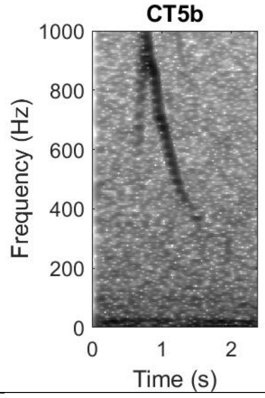	- tonal<br>- Fundamental 100-1200Hz<br>- 0.3-1.2s<br>- Downsweep<br>- Sometimes up-downsweep, sometimes upsweep, sometimes almost pulsed
CT6 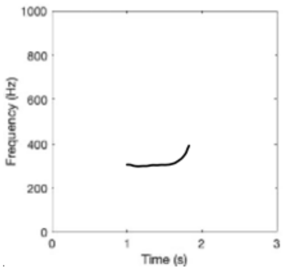	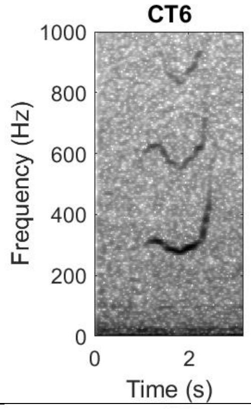	- tonal<br>- Fundamental 200-600Hz<br>- 1-2s<br>- Constant-upsweep<br>- Sometimes almost upsweep
CT8 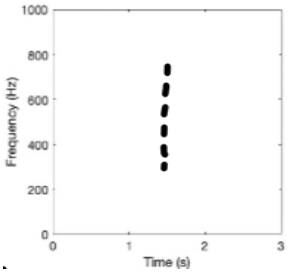	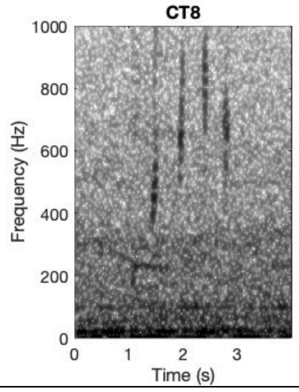	- Pulsed<br>- 300->2500Hz<br>- 0.1-0.3s<br>- Amplitude modulated<br>- Sometimes almost upsweep

CT10<br>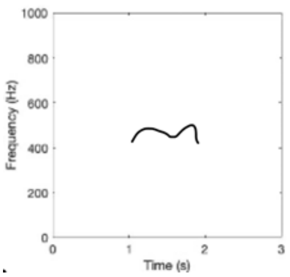	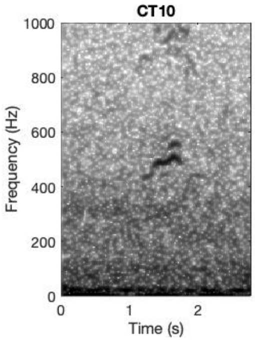	- tonal<br>- Fundamental 400-600Hz<br>- 0.5-2s<br>- Irregular frequency modulations<br>- Sometimes almost broadband
CT12<br>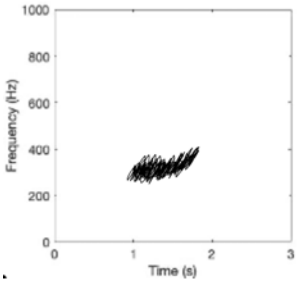	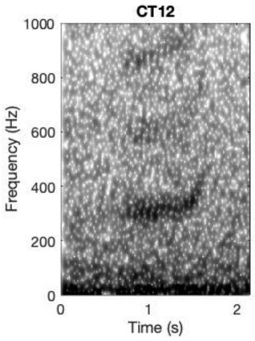	- Pulsed<br>- 200-500Hz<br>- 0.5-2s<br>- Broadband with increasing frequency<br>- Sometimes with ‘harmonics’ (Amplitude modulated)
CT18<br>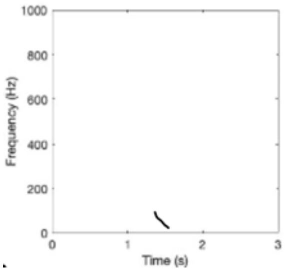	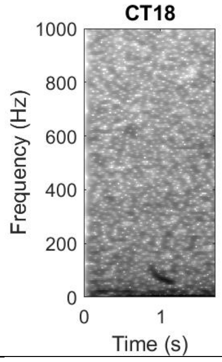	- tonal<br>- Fundamental 20-120Hz<br>- 0.2-1s<br>- Downsweep<br>- Sometimes up-downsweep
CT19<br>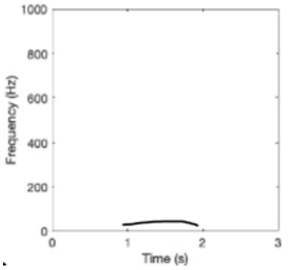	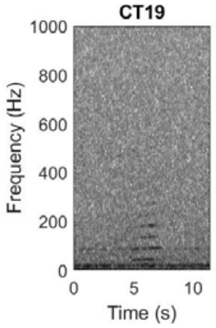	- tonal<br>- Fundamental 20-100Hz<br>- 1-4s<br>- Constant frequency<br>- Sometimes rather up-downsweep, down-upsweep, downsweep or upsweep

- ➔ Each phrase type is characterized by a distinct combination of units.
- ➔ Each phrase type is named by a capital letter and a lowercase letter, where the capital letter is an indicator for the first unit of the phrase type: i.e., Aa and Ab both start with call type (CT) 1.
- ➔ A phrase type is divided into phrase subtypes, when the number of repetitions of respective units differs: i.e., 1x CT1 and 3x CT4 translate into Aa13.

Type Aa

The combination of units CT1 & CT4(a)

Example of Aa13	
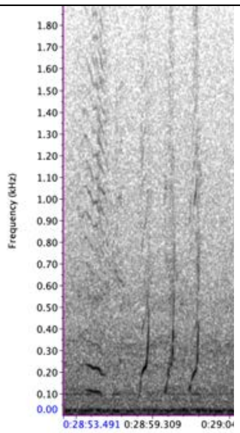	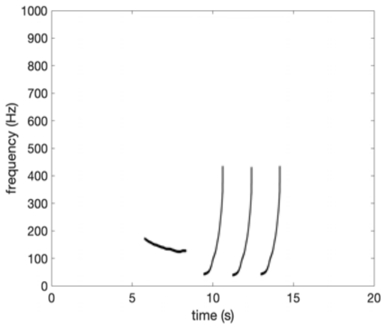

Type Ab

Combination CT1, CT4(a) & CT5(b)

Example of Ab123

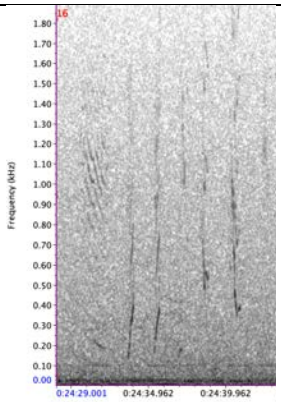

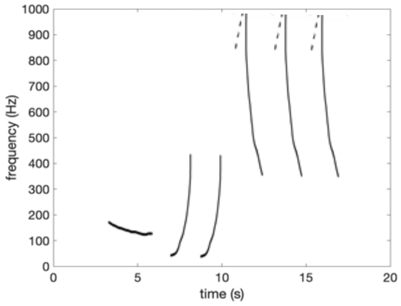

Type Ac

Combination of units CT1 & CT5(b)

Example of Ac13

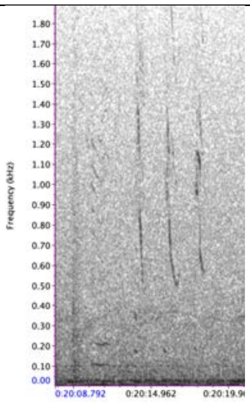

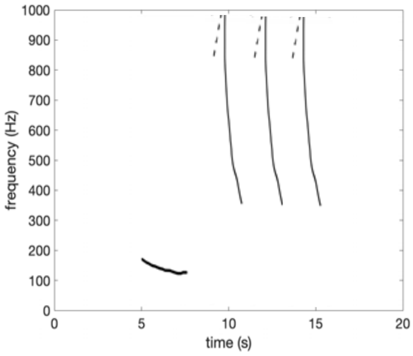

Type Ad

Combination of units CT1, CT5b, CT4a

Example of Ad123

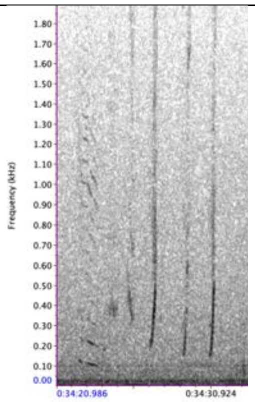

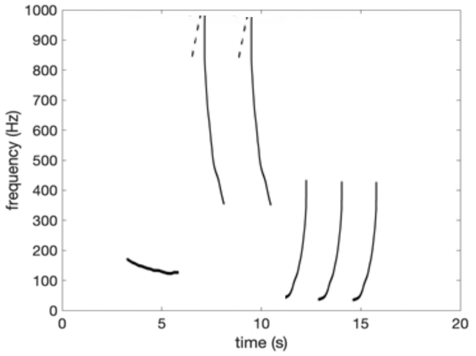

Type Ae

Combination of units CT1, CT6, CT5(b)

Example of Ae114

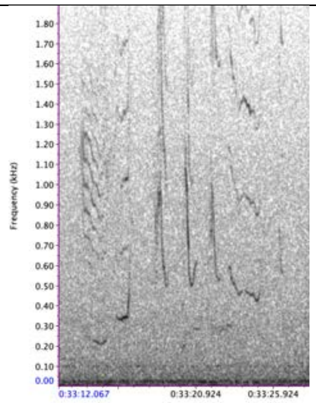

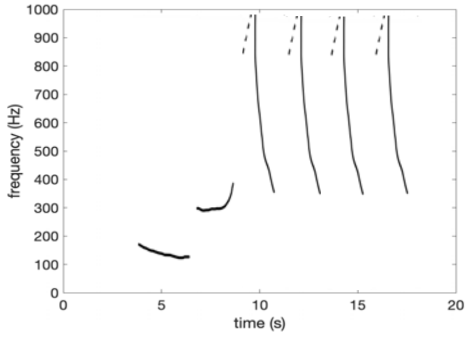

Type Af

Combination of units CT1, CT12, CT18

Example of Af112

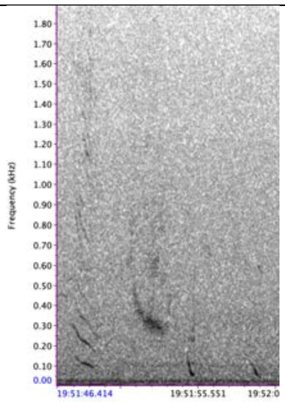

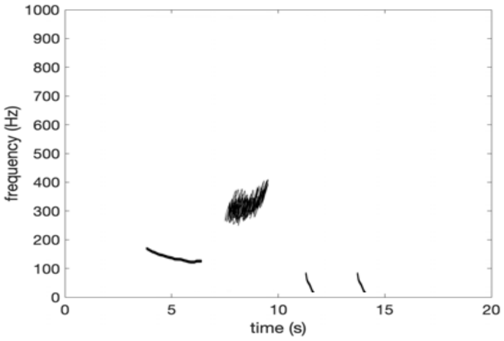

Type Ag

Combination of units CT1 and CT18

Example of Ag11

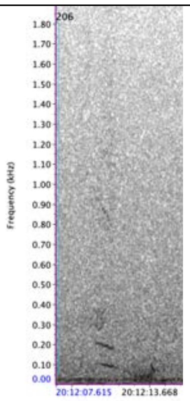

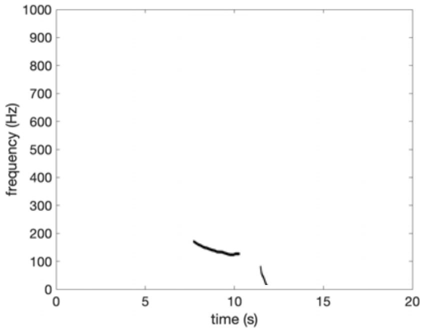

Type Ah

Combination of units CT1, CT12, CT4(a)

Example of Ah112	
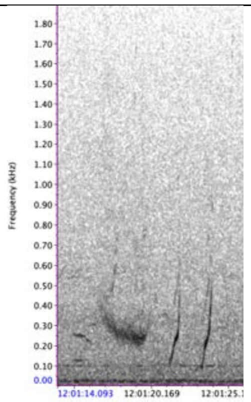	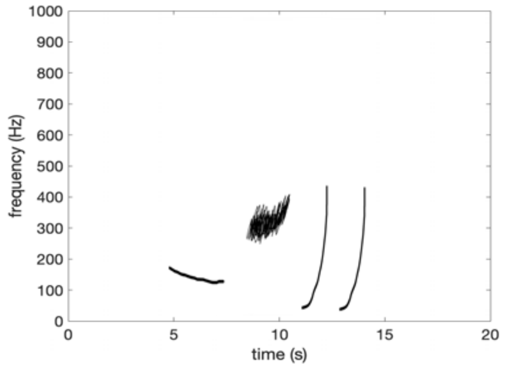

Type Ai

Combination of units CT1 and CT12

Example of Ai11	
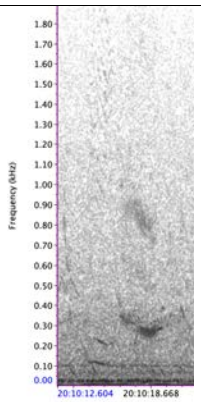	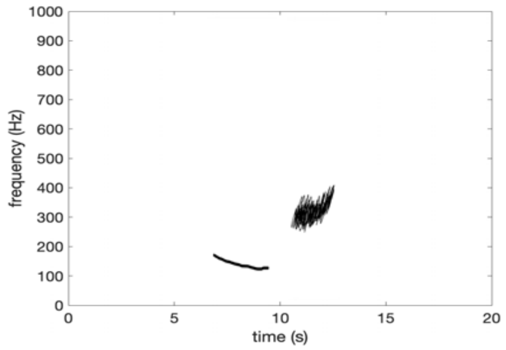

Type Aj

Combination of units CT1, CT12, CT5(b)

Example of Af114

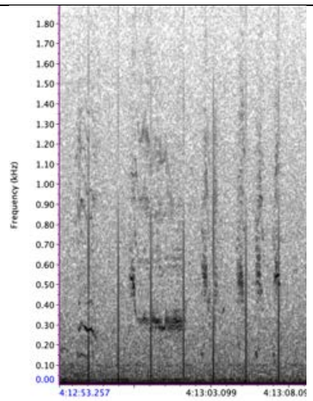

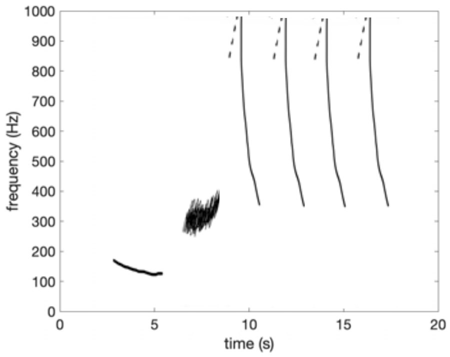

Type Ak

Combination of units CT1 & CT8

Example of Ak120

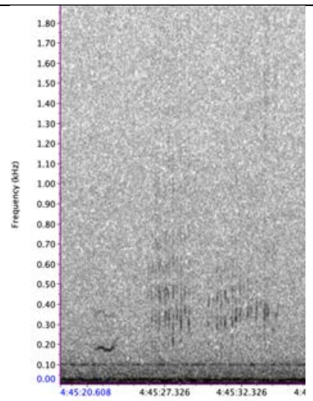

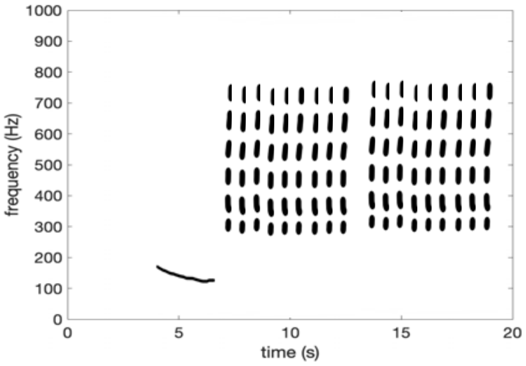

Type Al

Combination of units CT1, CT8, CT4(a)

Example of Al1171

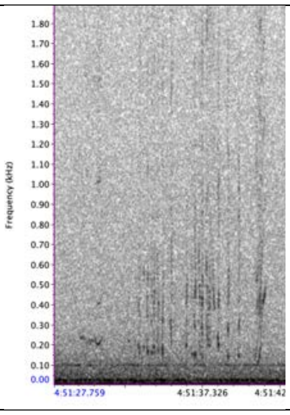

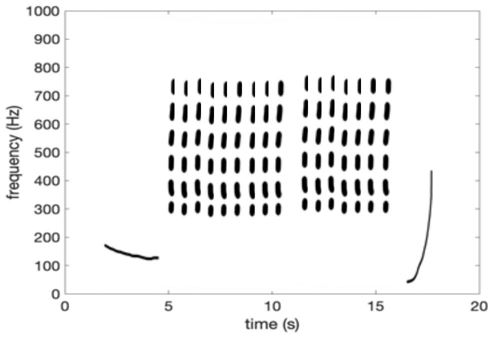

Type Am

Combination of units CT1, CT4(a), CT12

Example of Am122

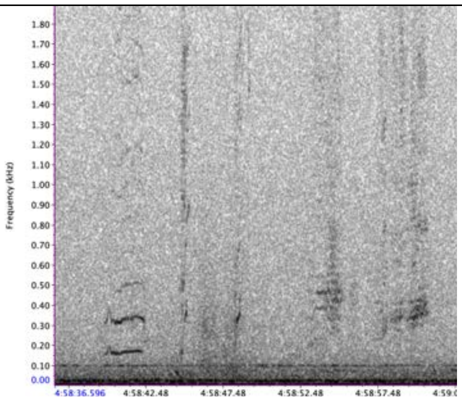

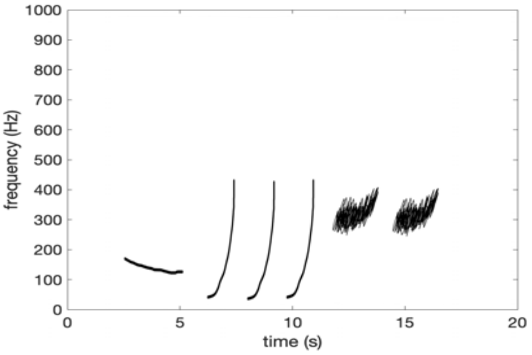

Type An

Combination of units CT1, CT12, CT8

Example of An1116	
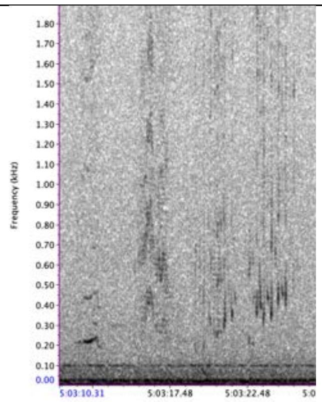	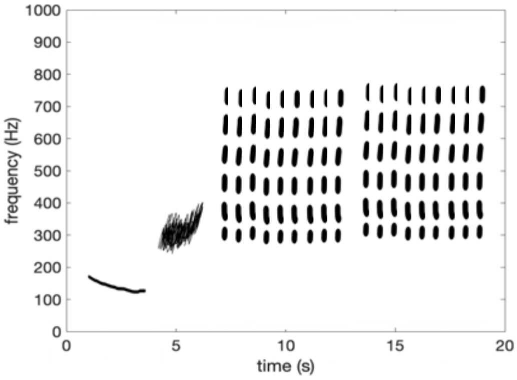

Type Ao

Combination of units CT1, CT5(b) & CT12

Example of Ao111	
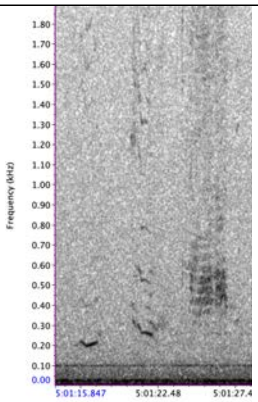	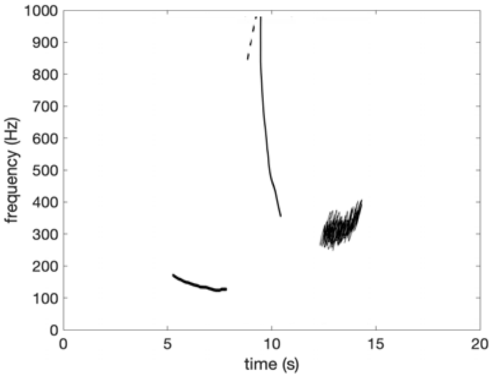

Type Ap

Combination of units CT1, CT4(a) & CT18

Example of Ap11214	
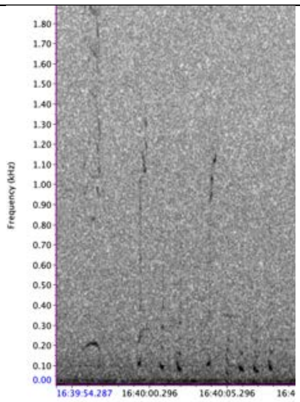	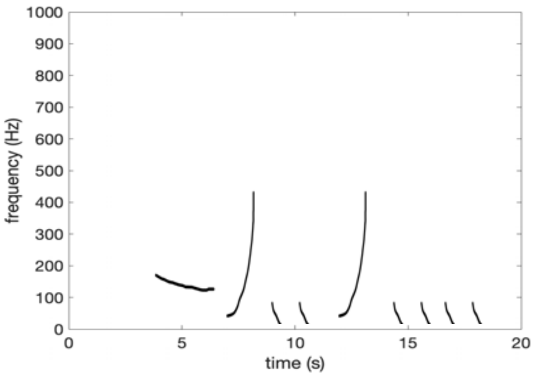

Type Aq

Combination of units CT1, CT5(b) & CT18

Example of Aq112121211	
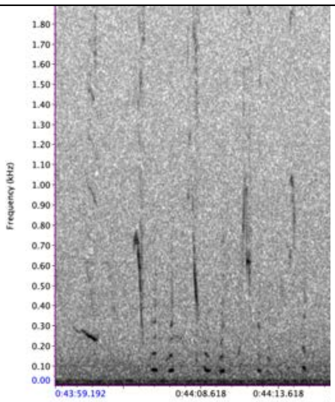	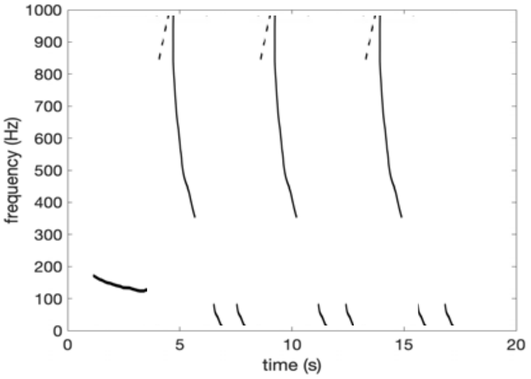

Type Ba

The combination of units CT6 & CT5(b)

Example of Ba17	
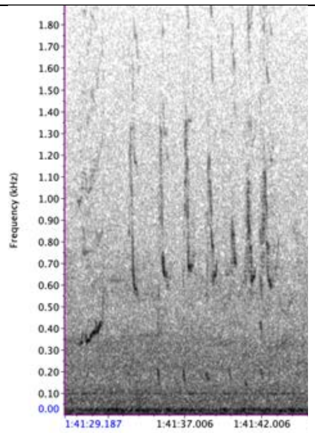	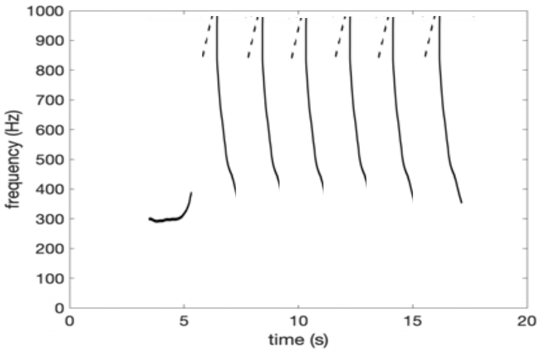

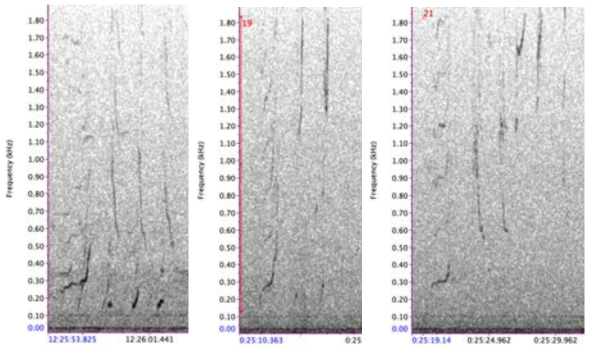

Type Bb

Combination of units CT6 & CT4(a)

Example of Bb12	
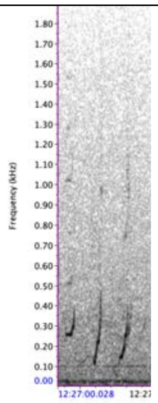	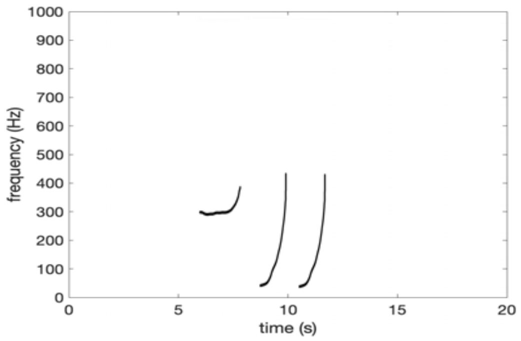

Type Bc

Combination of units CT6 & CT12

Example of Bc12	
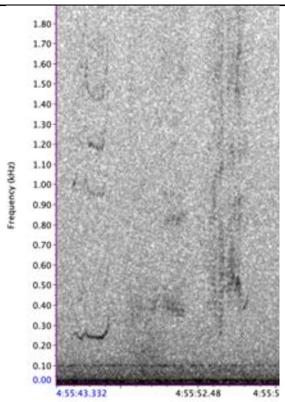	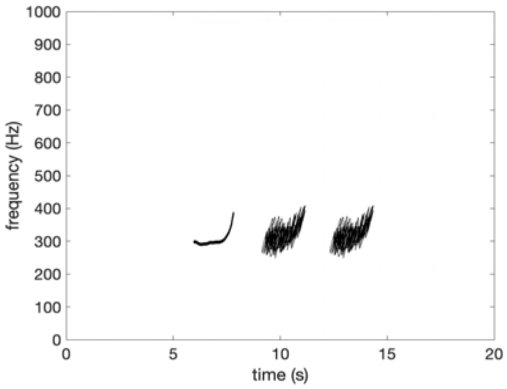

Type Bd

Combination of units CT6, CT4(a), CT10 & CT4(b)

Example of Bd2111	
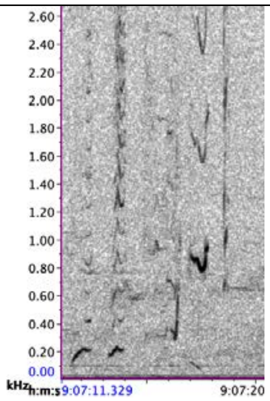	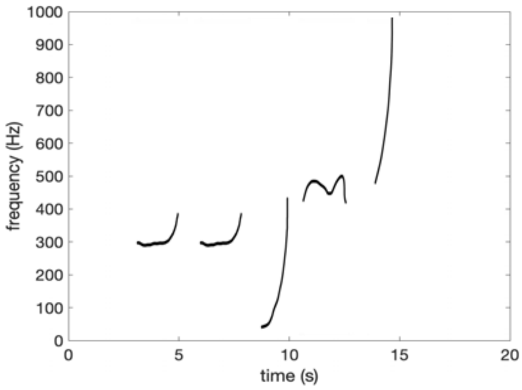

Type Be

Combination of units CT6, CT10

<table>
<tr><th colspan="2">Example of Be11</th></tr>
<tr><td>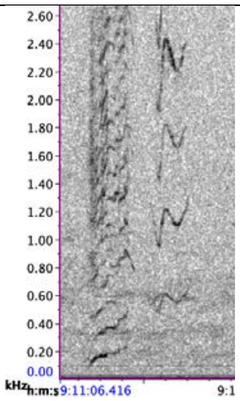
</td><td>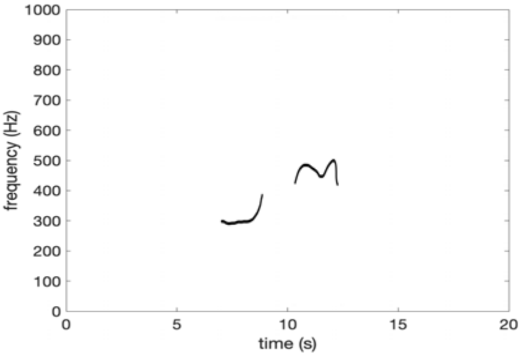
</td></tr>
</table>

Type Bf

Combination of units CT6, CT4(a), CT10

<table>
<tr><th colspan="2">Example of Bf211</th></tr>
<tr><td>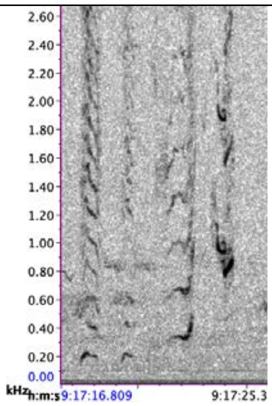
</td><td>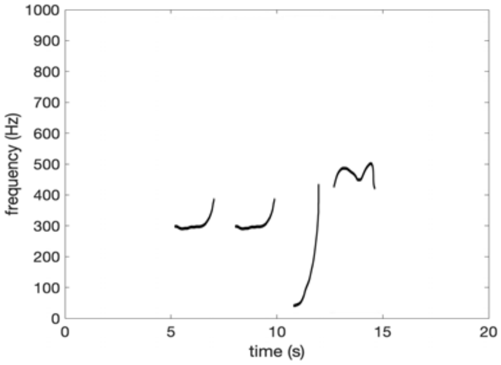
</td></tr>
</table>

Type Bg

Combination of units CT6, CT10, CT4(b)

Example of Bg111	
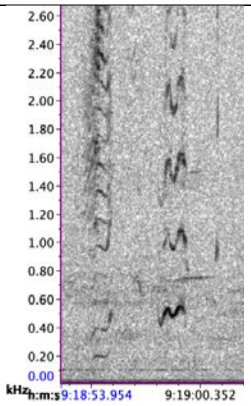	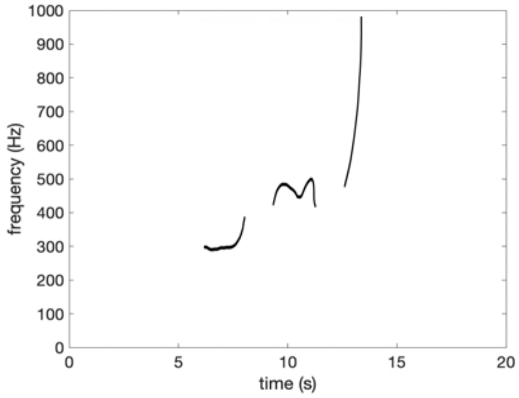

Type Bh

Combination of units CT6, CT1

Example of Bh13	
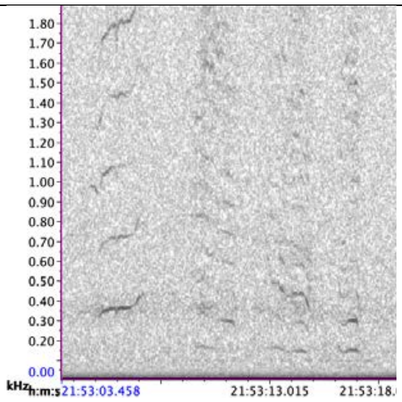	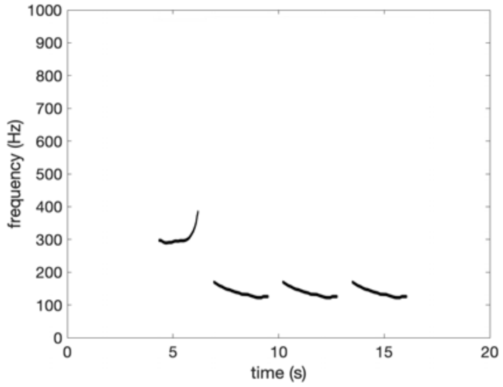

Type Bi

Combination of units CT6, CT1, CT4(a)

Example of Bi143

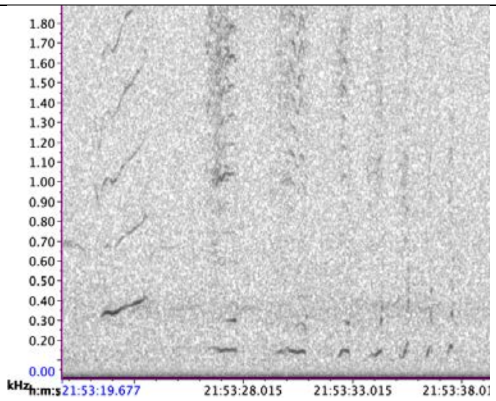

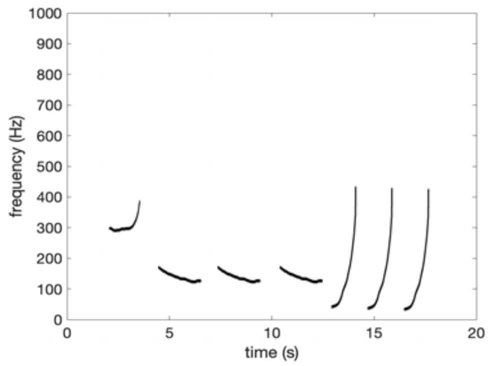

Type Bj

Combination of units CT6, CT12, CT4(a)

Example of Bj123

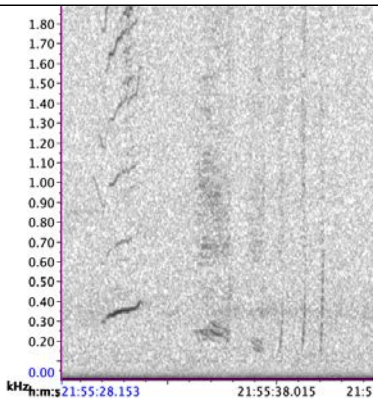

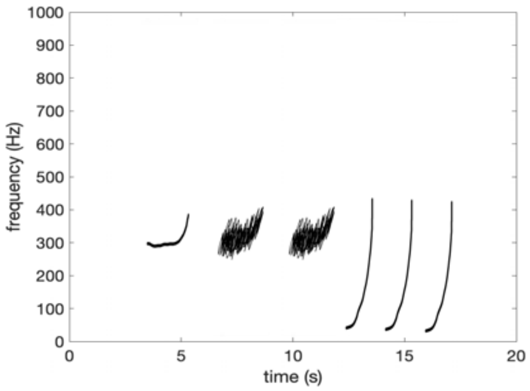

Type Bk

Combination of units CT6, CT18

Example of Bk15

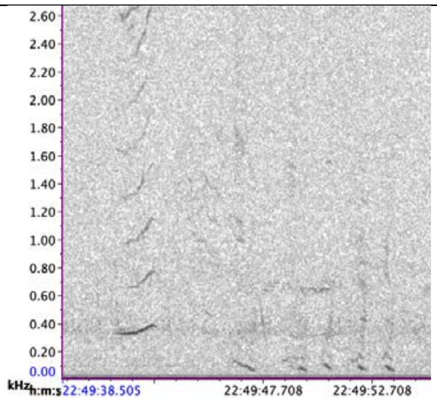

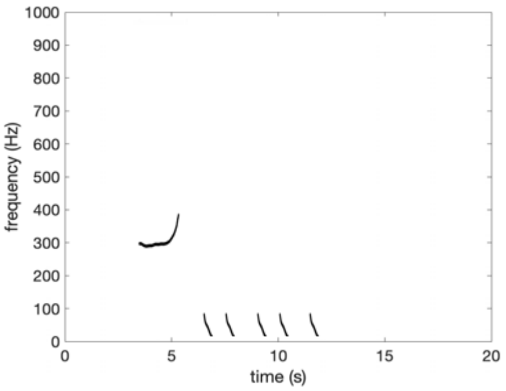

Type Ca

Combination of units CT3 & CT5(b)

Example of Ca13

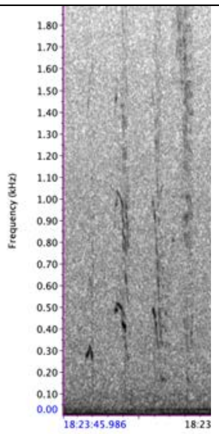

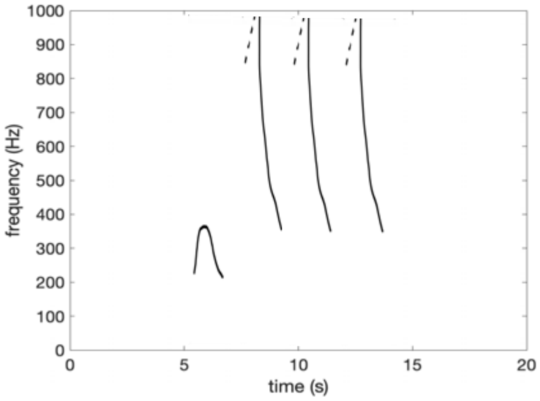

Type Cb

Combination of units CT3, CT18

Example of Cb11

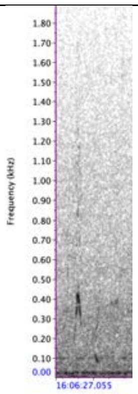

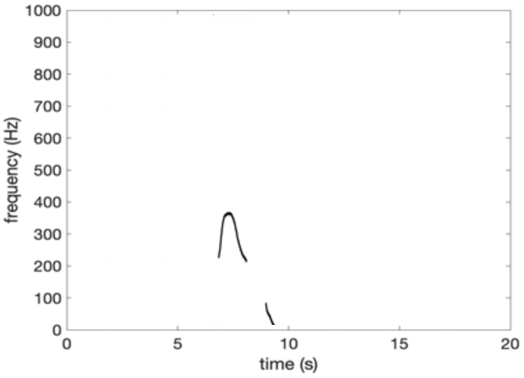

Type Cc

Combination of units CT3 and CT10

Example of Cc12

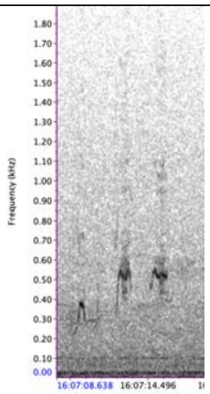

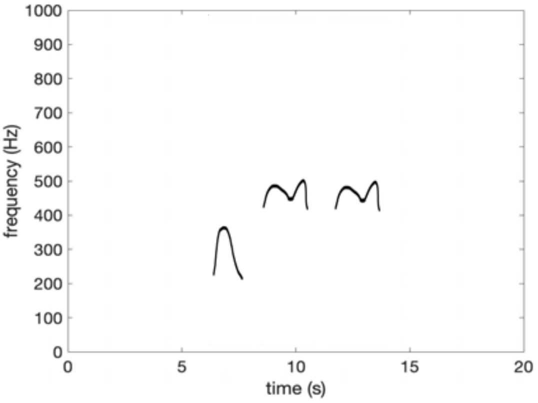

Type Cd

Combination of units CT3, CT18, CT10

Example of Cd111	
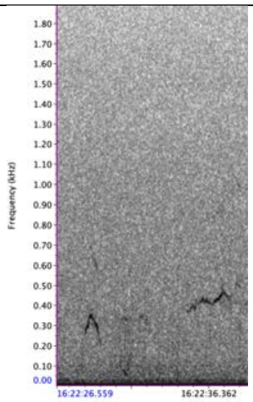	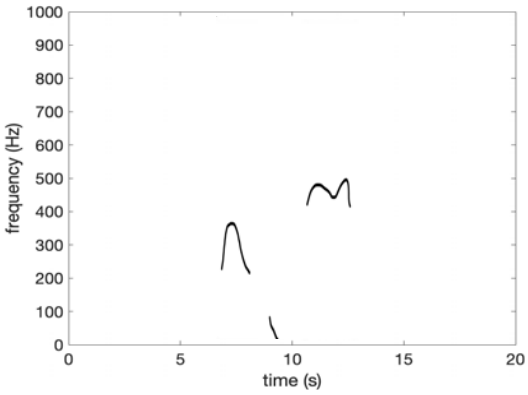

Type Ce

Combination of units CT3, CT10, CT18

Example of Ce111	
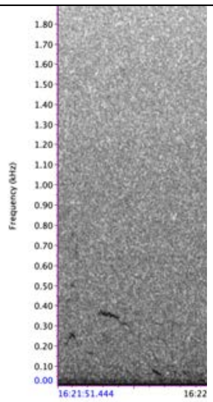	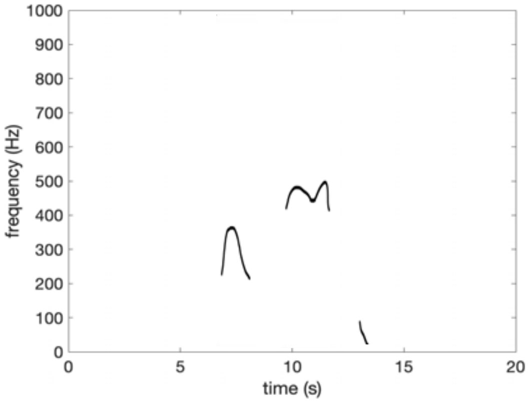

Type Cf

Combination of units CT3, CT10, CT5(b)

<table>
<tr><th colspan="2">Example of Cf115</th></tr>
<tr><td>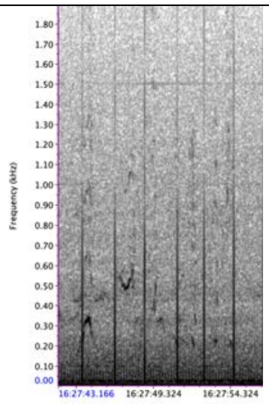
</td><td>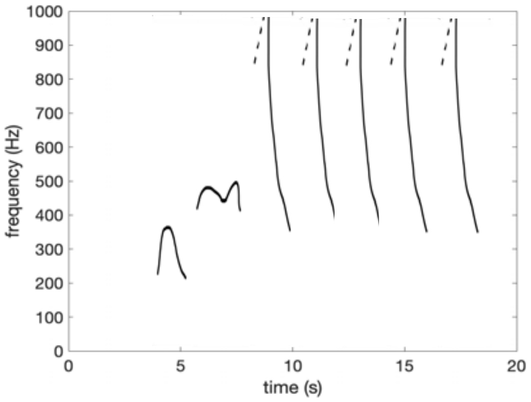
</td></tr>
</table>

Type Da

Combination of units CT12 and CT18

<table>
<tr><th colspan="2">Example of Da12</th></tr>
<tr><td>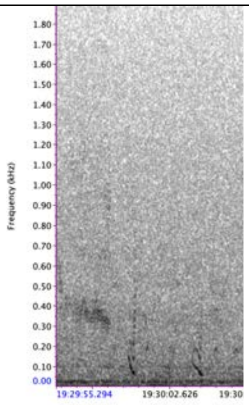
</td><td>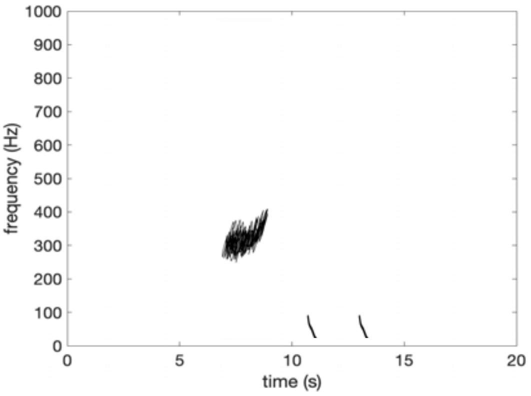
</td></tr>
</table>

Type Db

Combination of units CT12 and CT5(b)

Example of Db13	
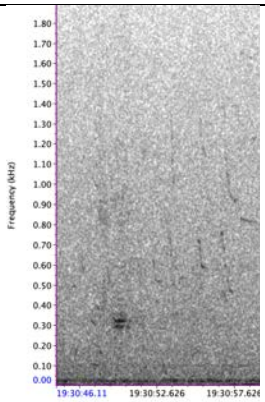	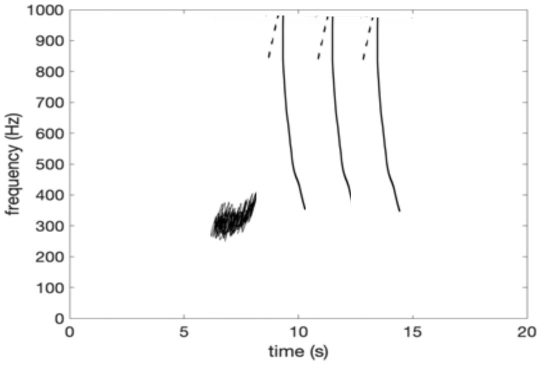

Type Dc

Combination of units CT12, CT4(a)

Example of Dc13	
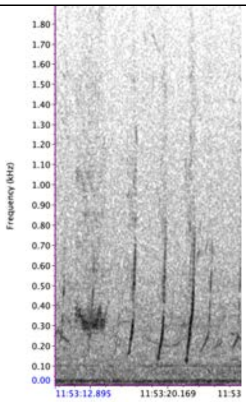	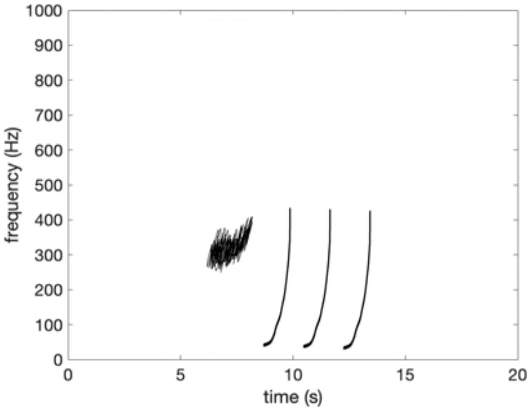

Type Dd

Combination of units CT12, CT1, CT4(a)

Example of Dd112	
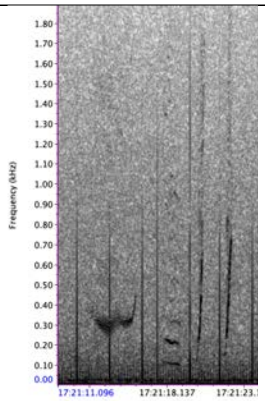	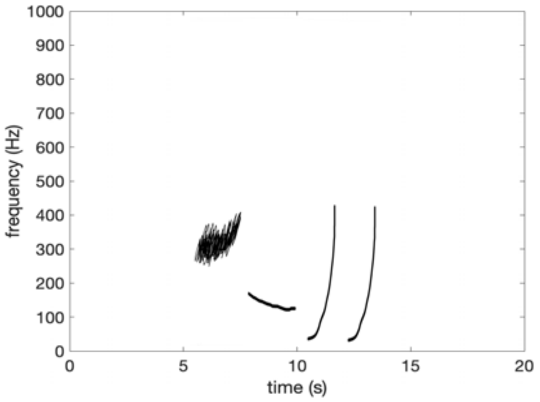

Type De

Combination of units CT12, CT18, CT5(b)

Example of De116	
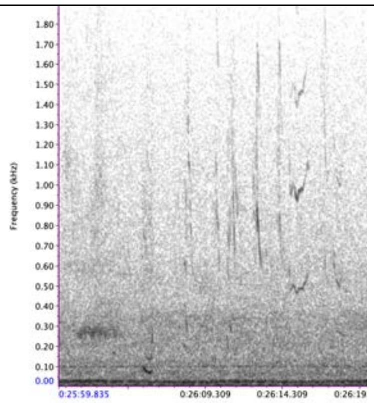	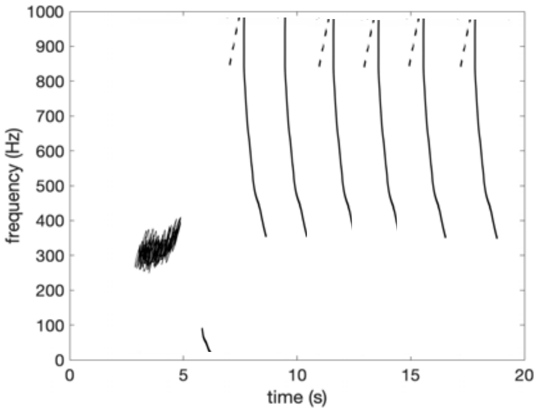

Type Df

Combination of units CT12, CT10

Example of Df11

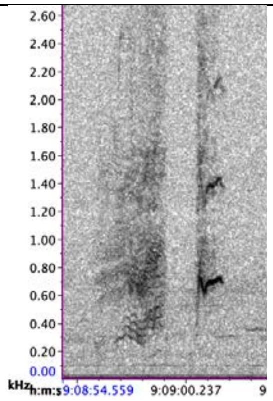

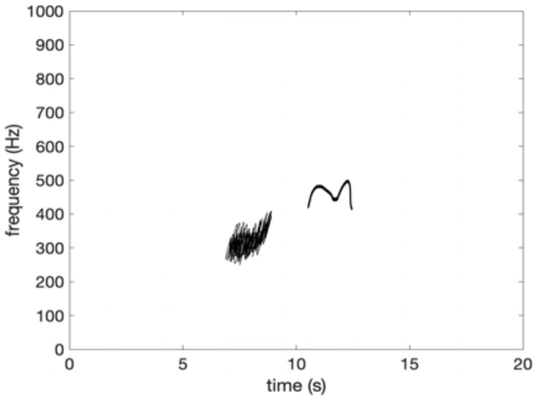

Type Dg

Combination of units CT12, CT10, CT4(b)

Example of Dg111

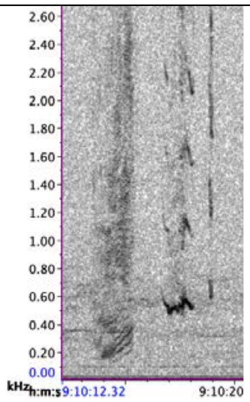

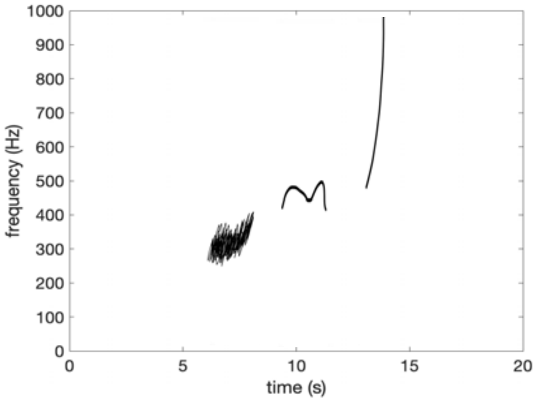

Type Dh

Combination of units CT12, CT10, CT4(b), CT4(a)

Example of Dh1111

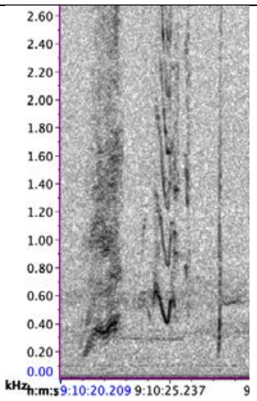

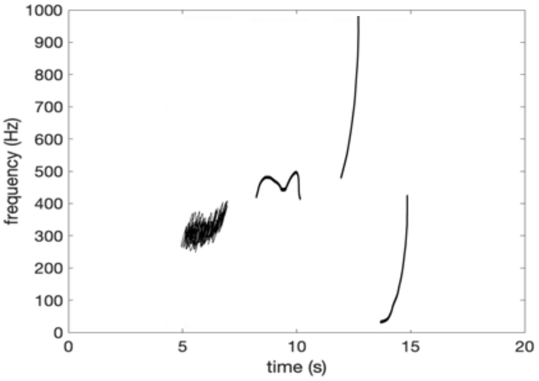

Type Ea

Combination of units CT4(a), CT8

Example of Ea13

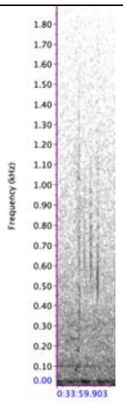

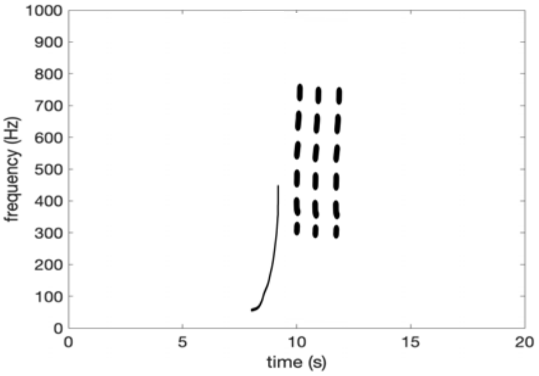

Type Eb

Combination of units CT4(a), CT5(b)

Example of Eb28

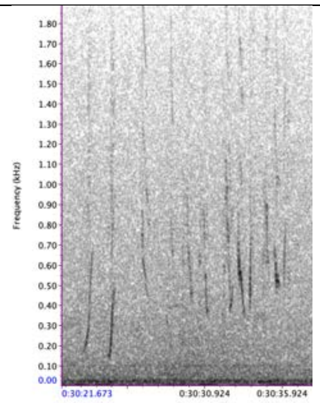

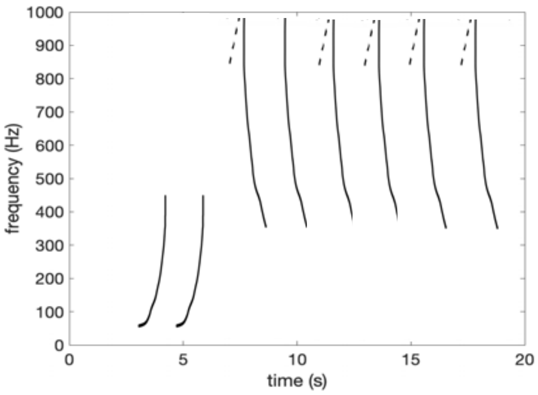

Type Ec

Combination of units CT4(b), CT5(b)

Example of Ec24

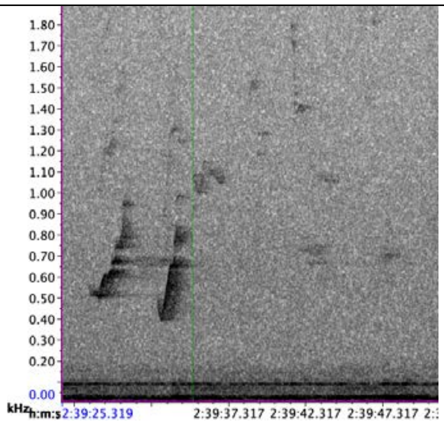

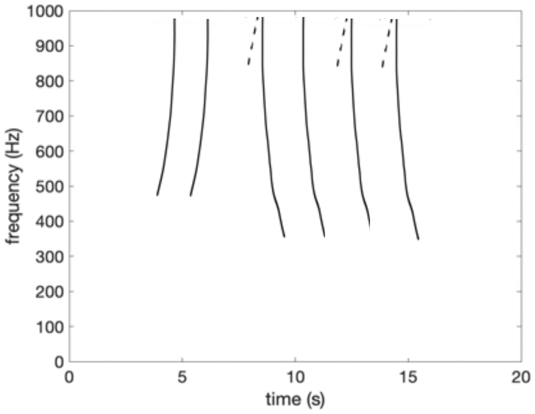

Type Ed

Combination of units CT4(b), CT1

Example of Ed31

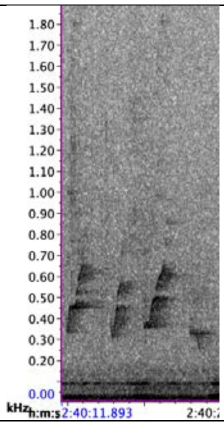

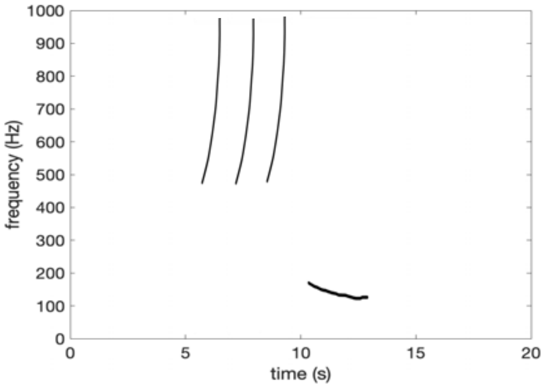

Type Ee

Combination of units CT4(a), CT4(b)

Example of Ee11

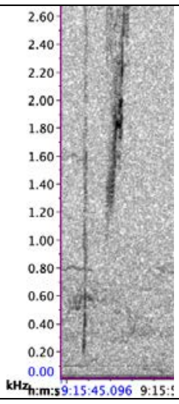

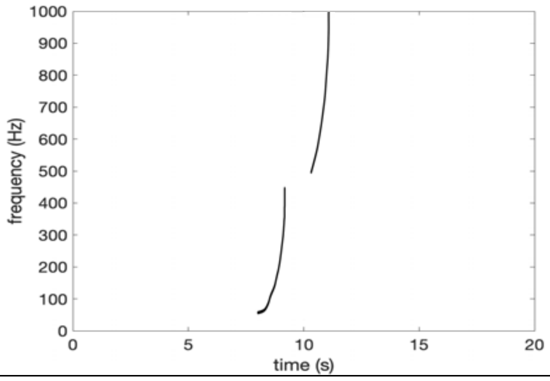

Type Ef

Combination of units CT4(a), CT6

Example of Ef11

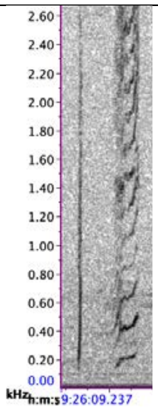

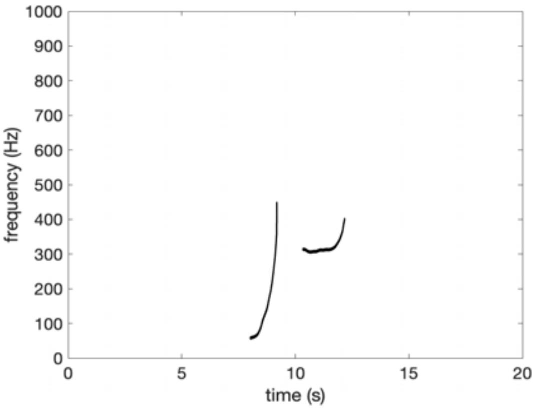

Type Eg

Combination of units CT4(a), CT6, CT4(b)

Example of Eg111

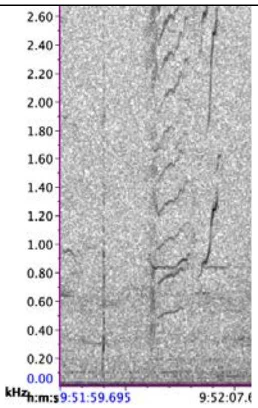

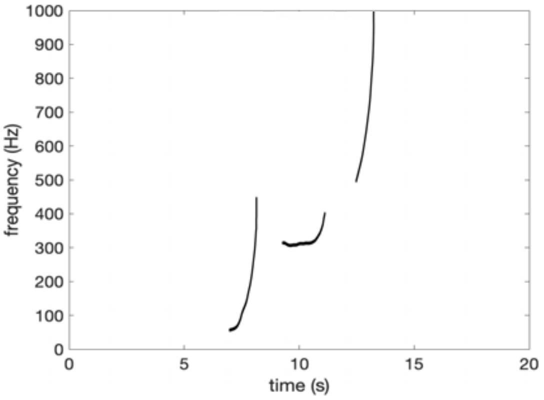

Type Fa

Combination of units CT18, CT5(b)

Example of Fa16

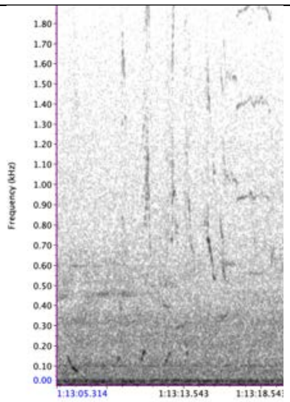

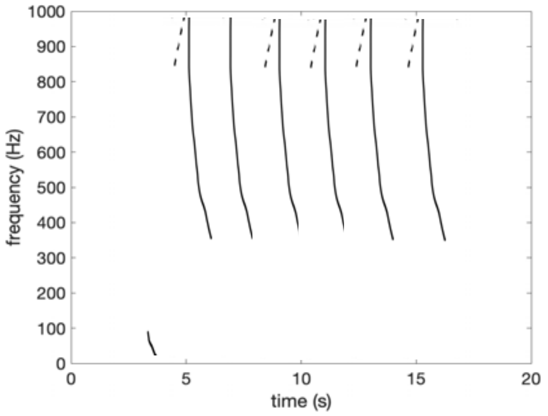

Type Ga

Combination of units CT5(a), CT19

Example of Ga11

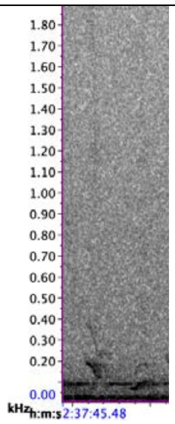

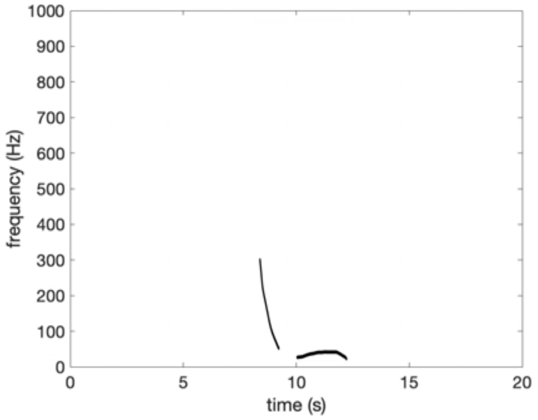

Type Gb

Combination of units CT5(a), CT1

Example of Gb11	
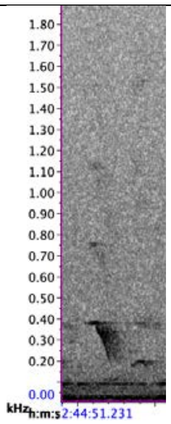	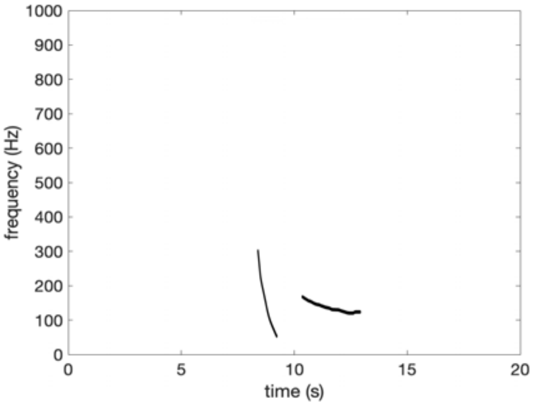

Type Gc

Combination of units CT5(a), CT4(a)

Example of Gc11	
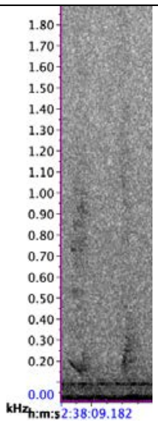	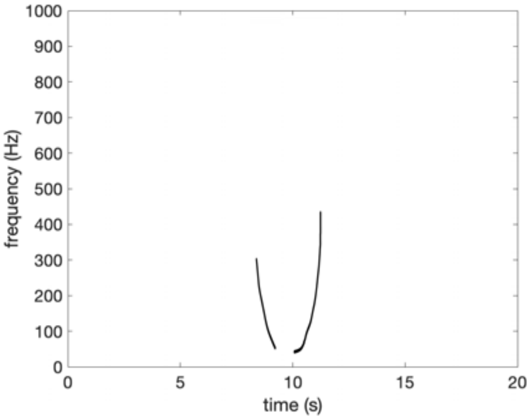

Type Gd

Combination of units CT5(b), CT5(a)

<table>
<tr><th colspan="2">Example of Gd12</th></tr>
<tr><td>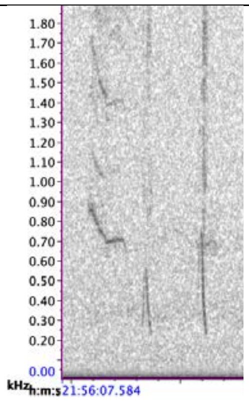
</td>
<td>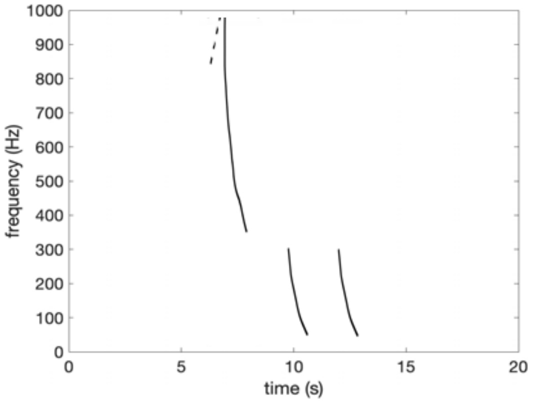
</td></tr>
</table>

Type Ge

Combination of units CT5(b), CT8

<table>
<tr><th colspan="2">Example of Ge111</th></tr>
<tr><td>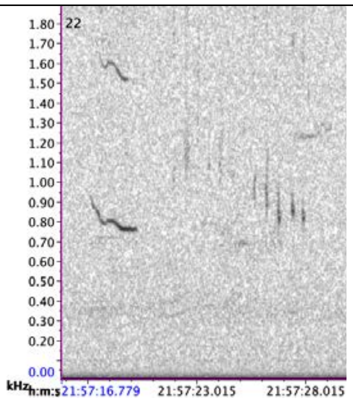
</td>
<td>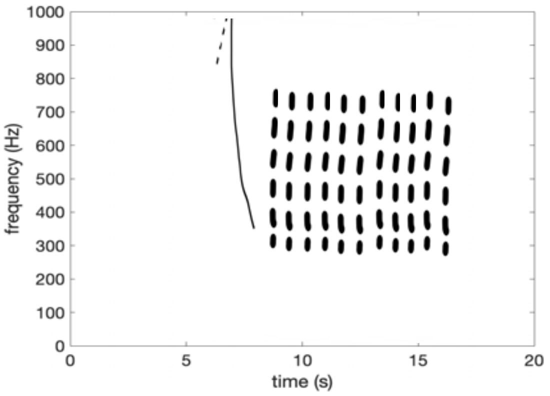
</td></tr>
</table>

Type Gf

Combination of units CT5(b), CT5(a), CT8

Example of Gf1113	
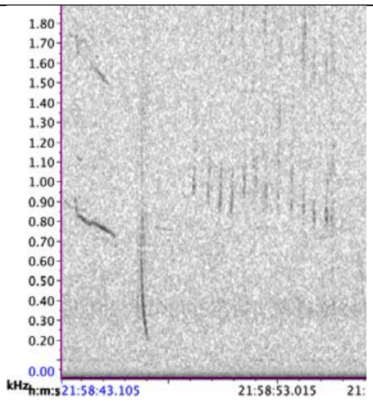	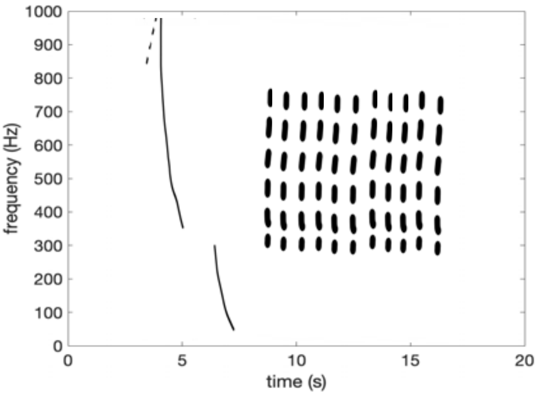

Type Gg

Combination of units CT5(b)/(a)

Example of Gg4	
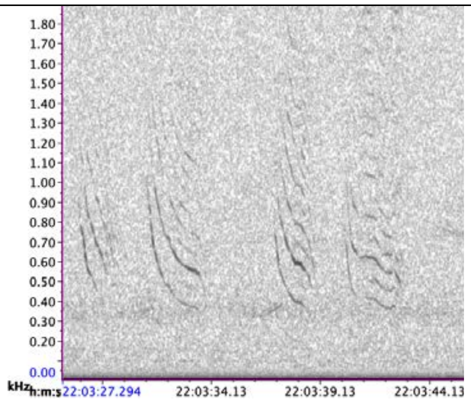	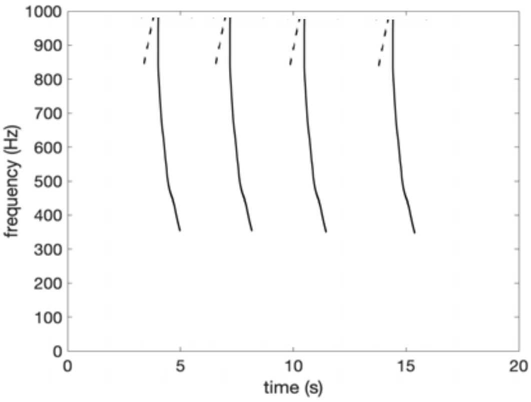

Type Gh

Combination of units CT5(b), CT4(a)

Example of Gh27

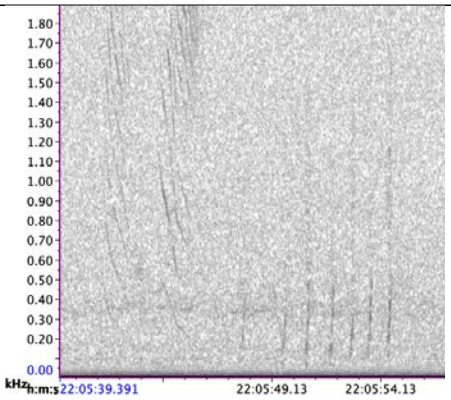

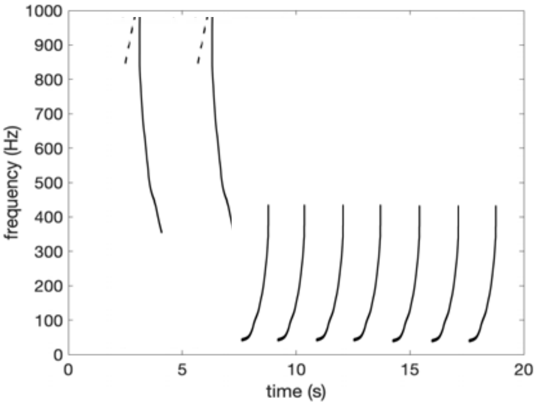

Type Gi

Combination of units CT5(b), CT12

Example of Gi12

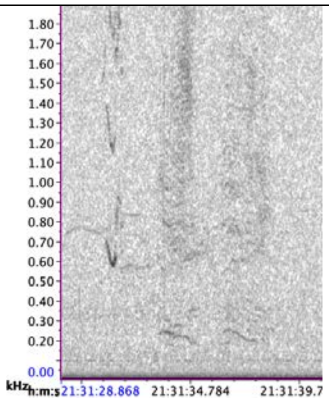

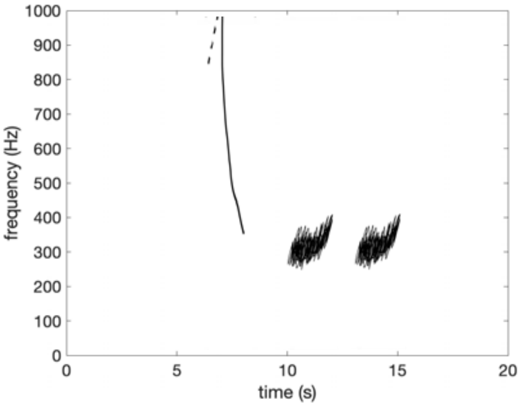

Type Ha

Combination of units CT19, CT4(a)

Example of Ha11

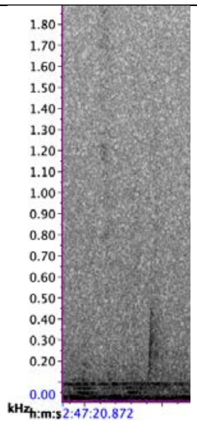

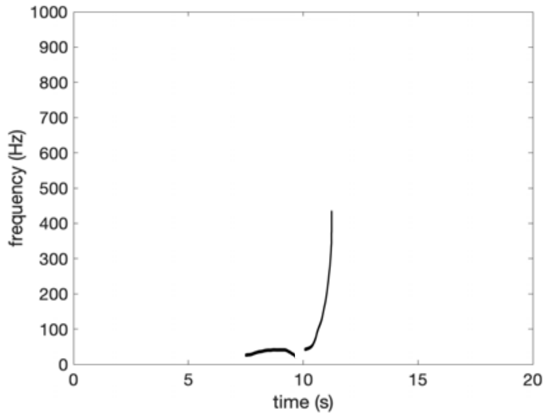

Type Ia

Combination of units CT10

Example of Ia5

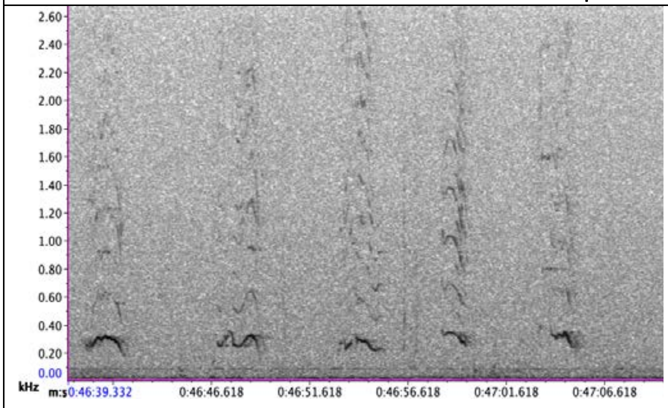

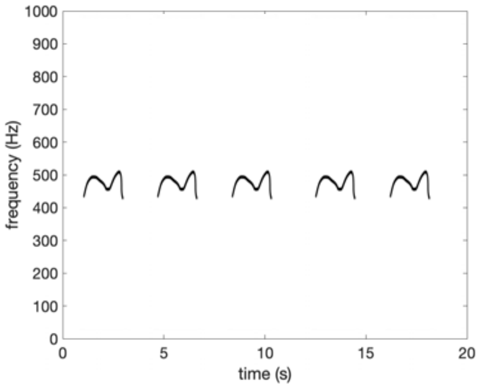

Type Ib

Combination of units CT10, CT8

Example of Ib12	
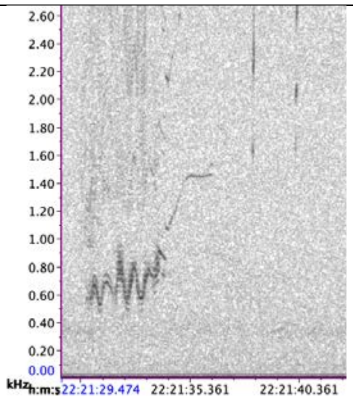	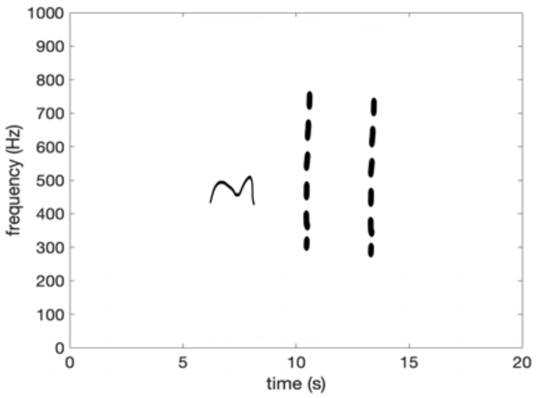
